# Genomic and Evolutionary Analysis of Two *Salmonella enterica* Serovar Kentucky Sequence Types Isolated from Bovine and Poultry Sources in North America

**DOI:** 10.1371/journal.pone.0161225

**Published:** 2016-10-03

**Authors:** Bradd J. Haley, Seon Woo Kim, James Pettengill, Yan Luo, Jeffrey S. Karns, Jo Ann S. Van Kessel

**Affiliations:** 1 Environmental Microbial and Food Safety Laboratory, Beltsville Area Research Center, Agricultural Research Services, United States Department of Agriculture, Beltsville, MD, United States of America; 2 Office of Analytics and Outreach, Center for Food Safety and Applied Nutrition, U.S. Food and Drug Administration, College Park, MD, United States of America; Institut National de la Recherche Agronomique, FRANCE

## Abstract

*Salmonella enterica* subsp. *enterica* serovar Kentucky is frequently isolated from healthy poultry and dairy cows and is occasionally isolated from people with clinical disease. A genomic analysis of 119 isolates collected in the United States from dairy cows, ground beef, poultry and poultry products, and human clinical cases was conducted. Results of the analysis demonstrated that the majority of poultry and bovine-associated *S*. Kentucky were sequence type (ST) 152. Several bovine-associated (n = 3) and food product isolates (n = 3) collected from the United States and the majority of human clinical isolates were ST198, a sequence type that is frequently isolated from poultry and occasionally from human clinical cases in Northern Africa, Europe and Southeast Asia. A phylogenetic analysis indicated that both STs are more closely related to other *Salmonella* serovars than they are to each other. Additionally, there was strong evidence of an evolutionary divergence between the poultry-associated and bovine-associated ST152 isolates that was due to polymorphisms in four core genome genes. The ST198 isolates recovered from dairy farms in the United States were phylogenetically distinct from those collected from human clinical cases with 66 core genome SNPs differentiating the two groups, but more isolates are needed to determine the significance of this distinction. Identification of *S*. Kentucky ST198 from dairy animals in the United States suggests that the presence of this pathogen should be monitored in food-producing animals.

## Introduction

*Salmonella enterica* subsp. *enterica* is a major cause of human and animal salmonellosis worldwide. The majority of human salmonellosis in the United States is caused by several serovars, namely Enteritidis, Typhimurium, Newport, and Javiana [1]. Although it is currently assumed that all serovars are potentially pathogenic to humans, the association between *S*. *enterica* and non-human animal illness as well as aymptomatic carriage is not as clear and frequently animals shedding several serovars such as Kentucky, Enteritidis, and Seftenberg are asymptomatic [1][2][3]. *S*. Kentucky is frequently isolated from both asymptomatic cattle, poultry and poultry products in the United States, but has been isolated from other sources such as the environment and domesticated dogs [2][3][4][5][6];;. Recently, the global spread of multi-drug resistant *S*. Kentucky ST198 has been described, indicating this serovar is an emerging public health threat[7]. In North America, human clinical cases of *S*. Kentucky ST198 infections have been associated with travel to the Middle East, Southeast Asia or Africa [7][8][9], and clinical cases caused by *S*. Kentucky ST152 are relatively rare.

*Salmonella* Kentucky has been identified as the most frequently isolated serovar from non-human non-clinical cases in the United States [1] and until recently, has mostly been considered a concern with poultry due to its high prevalence in broilers as well as the established link between poultry products and human salmonellosis. Further, *S*. Kentucky isolated from poultry and poultry products in the United States has been reported to be resistant to multiple antibiotics [4][10][11][12][13]. This serovar is currently the most frequently isolated serovar from poultry in the United States, recently supplanting Enteritidis and Heidelberg in poultry flocks [2]. In recent years, research on dairy farms has demonstrated that *S*. Kentucky is frequently isolated from dairy cows and dairy production operations in the United States and the isolation rates of this serovar in dairy cows appear to be increasing [3][14][15]). What remains unclear is how *S*. Kentucky is capable of colonizing these two distantly related hosts and if there are genomic differences between poultry-associated and bovine-associated *S*. Kentucky.

Both *S*. Kentucky ST152 and ST198 have been isolated globally, but the degree of relatedness among *S*. Kentucky isolates recovered on a global scale is not yet known. Multi-Locus Sequence Typing (MLST) analyses have demonstrated that there are no common alleles between these two STs, indicating that they are distantly related [7]. However, comprehensive description of the genomic differences between these two STs has not yet been conducted, and a comparison of the two may identify genomic factors involved in host-specificity and virulence potential. In a whole-genome phylogenetic analysis of *S*. *enterica* Timme et al. [16] demonstrated that *S*. Kentucky was polyphyletic; a phenomenon identified in other serovars [17]. For *S*. *enterica* and other bacteria, polyphyly has been associated with lateral gene transfer (LGT) of the antigenic coding regions [17][18]. The historical application of serology to strain naming and description has thus been misleading in that distantly related strains that have acquired an antigenic coding region from the same source through LGT have been presumed to be similar based on this scheme, when in fact their genomes may be highly diverged.

The objective of this study was to infer the phylogeny of *S*. Kentucky, identify the genomic features associated with the apparent specificity of *S*. Kentucky ST152 to bovine and poultry hosts/products, and to identify differences between *S*. Kentucky ST152 and ST198 genomes. To accomplish this we sequenced the genomes of 49 dairy-cow associated isolates and two poultry-associated isolates that were collected from across the United States, and coupled these data with the genomes of 68 isolates previously collected from poultry, poultry products and production environments and clinical cases in the United States, Canada, and the Middle East.

## Materials and Methods

*Salmonella* Kentucky isolates from dairy cows were recovered from previously collected National Animal Health Monitoring System (NAHMS) samples as well as an eight-year dairy farm monitoring program conducted in south-central Pennsylvania [3][19] (two poultry-associated isolates were supplied by S. Parveen). Isolates were serogrouped following the methods of Herrera-Leon et al. [20]and Karns et al. [21]and serotypes were identified by National Veterinary Services Laboratories (NVSL; Ames, IA). Isolates were streaked onto tryptic soy agar, and a single colony was inoculated in tryptic soy broth overnight at 37°C. This inoculum was centrifuged, decanted, and then processed for DNA extraction using a Qiagen DNeasy Kit (Qiagen, Valencia, CA). Nextera XT libraries were made for each sample and pooled into equimolar concentrations following the manufacturer’s instructions (Illumina, San Diego, CA). Paired-end sequencing (2 X 151 bp) was conducted on an Illumina NextSeq 500 sequencing platform with a High-Output flow cell. Data were demultiplexed and trimmed to remove adaptor sequences using the BCL2FastQ program and PhiX reads were removed using DeconSeq [22]. Reads were further cleaned using Trimmomatic [23] and assembled using SPAdes 3.6.2 [24]. ST56 complex genomes were downloaded from NCBI GenBank prior to February 2016 and ST15 complex genomes were downloaded prior to December 2015 (PRJNA242614, PRJNA273513, PRJNA78335, PRJNA78339, PRJNA78337, PRJNA225734, PRJNA225734, PRJNA66693, PRJNA20069, PRJNA19457, PRJEB6491, PRJNA337914, and PRJNA186035) (S1 Table). Genomes from BioProject PRJEB6491 [25] were labeled as 915c and 917c in this analysis. Bovine-associated *S*. Kentucky isolates used in this study will be provided upon request.

Three methods were used to identify SNPs among the *S*. Kentucky genomes. To identify high quality SNPs based on read coverage, Lyve-SET [26] [27]and CFSAN SNP Pipeline [28] [29] were used. For the Lyve-SET analysis SNP identification was conducted with a minimum 10X coverage requirement and the CGP read cleaner. When fastq files were not available, assembled genomes were included in the project/asm directory prior to SNP detection. Default settings were used for the CFSAN SNP Pipeline analysis. For this analysis only genomes for which fastq files or.sff files were available could be used for the SNP search and therefore one ST152 (strain CDC 191) genome and several ST198 genomes were excluded (strains CVM 43824, CVM 43780, CVM 43756). To identify SNPs in assembled genomes, Parsnp from the Harvest package was used [30][31]. Regions undergoing high levels of recombination were removed using the–x option (PhiPack) and all genomes were included using the–c option. The chromosome of *S*. Kentucky CVM21988 was selected as the reference genome for all analyses. Parsnp was used to determine the core-genome SNP differences between *S*. Kentucky ST152 and ST198 genomes and other subclade A1 serovars identified by Timme et al. [16]. SNPs were annotated using snpEff [32].

To infer the phylogeny of *S*. Kentucky ST152/318/2132 isolates the multi-fasta SNP matrices from each of the three SNP detection methods were imported into MEGA6 [33] and RAxML [34]. Maximum Parsimony (MP) trees were inferred for all *S*. Kentucky isolates (ST198, ST152/318/2132, and unknown STs) as well as *S*. Kentucky isolates with non-*S*. Kentucky isolates using MEGA6. For phylogenetic inference of ST152/318/2132 isolates a Maximum Likelihood phylogeny was inferred using RAxML version 8.2.8 with the General Time Reversible (GTR) selected as the model of nucleotide substitution and all other parameters set to default settings. MP and ML analyses were each conducted with 1000 bootstrap replicates. The bootstrapped MP tree inferred from the Parsnp method of SNP detection was used to determine the genealogical sorting index (gsi) [35] for bovine and poultry isolates using the GSI webserver [36]. MLST data were retrieved from the University of Warwick *Salmonella enterica* MLST database [6]and through the Center for Genomic Epidemiology server [37].

The resulting SNP matrices were also analyzed using the Bayesian clustering program STRUCTURE v2.3.4 [38]. This program clusters individuals based on patterns of SNP differences without enforcing a bifurcating tree-like structure and, thus, may reveal additional details about the genomic content, similarity, and differences among isolates. In particular, STRUCTURE is suitable for detecting evidence for admixture (i.e., individuals whose genomes appear to be a combination of SNPs associated with distinct groups). STRUCTURE analyses were run with default settings and 60,000 generations, the first 10,000 of which served as the burnin. STRUCTURE results were visualized using DISTRUCT v1.1 [39].

Putative plasmid sequences were identified with PlasmidFinder [40] using the *Enterobacteriaceae* database with the detection thresholds set to 95% sequence identity. The identified HSP fragments were compared to the NCBI database (BLASTN analysis) to identify similar plasmids. The presence/absence of protein coding genes was conducted using BLASTP. Putative genomic islands in *S*. Kentucky CVM29188 (ST152) and *S*. Kentucky CVM N51290 (ST198) were identified using IslandViewer 3 [41].

Forty nine bovine-associated *S*. Kentucky isolates, and two poultry-associated isolates (including type strain ATCC 9263) were evaluated for their susceptibility to 25 antimicrobials (azithromycin, ciprofloxacin, gentamicin, tetracycline, nalidixic acid, cefoxitin, chloramphenicol, ceftriaxone, amoxicillin/clavulanic acid, ceftiofur, sulfisoxazole, trimethoprim/sulfamethoxazole, ampicillin, streptomycin, cephalothin, cefotaxime, cefotaxime/clavulanic acid, ceftazidime, ceftazidime/clavulanic acid, imipenem, cefepime, cefpodoxime, piperacillin/tazobactam, meropenem, and cefazolin) using an automated microdilution procedure (Sensititre, ThermoFisher, Lenexa, KS) and specialty plates CVM3AGNF and ESB1F. Antimicrobial minimum inhibitory concentrations (MICs) were interpreted based upon the epidemiological cut-off values (ECOFFs) used by the National Antimicrobial Resistance Monitoring System (NARMS).

## Results and Discussion

### Description of Isolates and Antibiotic Susceptibility Testing of Bovine-associated Isolates

In total 119 *S*. Kentucky genomes were utilized in the *in silico* analyses of this study. Of these, 49 dairy cow-associated isolates were selected from a collection of isolates that have been recovered from routine analysis of milk, milk filters, fecal samples from dairy cows, and the dairy cow farm environment in the United States over a 13-year period (Table 1). Two poultry isolates recovered from a broiler operation in the Eastern Shore of Maryland were used in this study, and the remaining poultry-associated genomes (n = 59) were gathered as assembled genomes and raw sequencing reads from the NCBI GenBank database and the SRA database, respectively. All human clinical isolates (n = 6) were all gathered from NCBI (two as raw sequencing reads and four as assembled genomes).

**Table 1:** Kentucky genomes used in this study. *S*.

Genome	Source	Location of Isolation	Year of Isolation	Sequence Type (ST)^1^	ST Complex ^2^	Phylogenetic Cluster ^3^
ARS-CC97	Fecal composite (dairy cow farm)	Pennsylvania (USA)	2004	318	15	1.2.2
0253	Dairy cow feces	Pennsylvania (USA)	2004	318	15	1.2.2
5349	Dairy cow feces	Pennsylvania (USA)	2006	152	15	1.2.3
ARS-CC444	Milk filter (dairy cow farm)	Wisconsin (USA)	2007	152	15	1.1
ARS-CC457	Milk filter (dairy cow farm)	Texas (USA)	2007	152	15	1.1
ARS-CC515	Bulk tank milk (dairy cow farm)	Wisconsin (USA)	2007	152	15	1.1
ARS-CC521	Milk filter (dairy cow farm)	Wisconsin (USA)	2007	152	15	1.1
ARS-CC572	Milk filter (dairy cow farm)	Wisconsin (USA)	2007	152	15	1.1
ARS-CC913	Dairy cow feces	Virginia (USA)	2007	152	15	1.2.1
ARS-CC526	Bulk tank milk (dairy cow farm)	Pennsylvania (USA)	2007	152	15	1.2.1
ARS-CC496	Milk filter (dairy cow farm)	New York (USA)	2007	152	15	1.2.3
CFSAN011775	Dairy cow feces	Pennsylvania (USA)	2008	152	15	1.2.3
CFSAN011776	Dairy cow feces	Pennsylvania (USA)	2009	152	15	1.2.3
CFSAN011777	Dairy cow feces	Pennsylvania (USA)	2009	152	15	1.2.3
CFSAN011778	Dairy cow feces	Pennsylvania (USA)	2009	152	15	1.2.3
CFSAN011779	Dairy cow feces	Pennsylvania (USA)	2010	152	15	1.2.3
CFSAN011780	Dairy cow feces	Pennsylvania (USA)	2010	152	15	1.2.3
CFSAN011782	Dairy cow feces	Pennsylvania (USA)	2011	152	15	1.2.3
ARS-CC6181	Milk filter (dairy cow farm)	Pennsylvania (USA)	2011	152	15	1.2.3
ARS-CC6183	Milk filter (dairy cow farm)	Pennsylvania (USA)	2011	152	15	1.2.3
ARS-CC6204	Milk filter (dairy cow farm)	Pennsylvania (USA)	2011	152	15	1.2.3
ARS-CC6329	Milk filter (dairy cow farm)	Pennsylvania (USA)	2011	152	15	1.2.3
ARS-CC6333	Milk filter (dairy cow farm)	Pennsylvania (USA)	2011	152	15	1.2.3
ARS-CC6340	Milk filter (dairy cow farm)	Pennsylvania (USA)	2011	152	15	1.2.3
ARS-CC8561	Dairy cow feces	Pennsylvania (USA)	2011	152	15	1.2.3
ARS-CC8574	Fecal composite (dairy cow farm)	Pennsylvania (USA)	2011	152	15	1.2.3
ARS-CC8601	Dairy cow feces	Pennsylvania (USA)	2011	152	15	1.2.3
ARS-CC8619	Dairy cow feces	Pennsylvania (USA)	2011	152	15	1.2.3
ARS-CC8624	Dairy cow feces	Pennsylvania (USA)	2011	152	15	1.2.3
ARS-CC8625	Dairy cow feces	Pennsylvania (USA)	2011	152	15	1.2.3
ARS-CC8633	Fecal composite (dairy cow farm)	Pennsylvania (USA)	2011	152	15	1.2.3
CFSAN011781	Dairy cow feces	Pennsylvania (USA)	2012	152	15	1.2.3
ARS-CC7487	Dairy cow hide swab	Pennsylvania (USA)	2013	152	15	1.2.3
ARS-CC8078	Fecal composite (dairy cow farm)	Pennsylvania (USA)	2013	152	15	1.2.3
ARS-CC8289	Milk filter (dairy cow farm)	Ohio (USA)	2014	152	15	1.2.2
ARS-CC8294	Milk filter (dairy cow farm)	Pennsylvania (USA)	2014	152	15	1.2.3
ARS-CC8297	Milk filter (dairy cow farm)	Ohio (USA)	2014	152	15	1.2.3
ARS-CC8417	Trough water (dairy cow farm)	Pennsylvania (USA)	2014	152	15	1.2.3
ARS-CC8446	Milk filter (dairy cow farm)	Ohio (USA)	2014	152	15	1.2.3
ARS-CC353	Milk filter (dairy cow farm)	New York (USA)	2007	152	15	2.1
ARS-CC469	Bulk tank milk (dairy cow farm)	Vermont (USA)	2007	152	15	2.1
ARS-CC661	Milk filter (dairy cow farm)	Vermont (USA)	2007	152	15	2.1
ARS-CC690	Milk filter (dairy cow farm)	Wisconsin (USA)	2007	152	15	2.1
ARS-CC912	Dairy cow feces	Wisconsin (USA)	2007	152	15	2.1
ARS-CC917	Dairy cow feces	Vermont (USA)	2007	152	15	2.1
CDC 191	Human clinical	Wisconsin (USA)	2002	152	15	2.1
ARS-CC621	Milk filter (dairy cow farm)	Texas (USA)	2007	152	15	2.2
CVM N50435	Chicken breast	New Mexico (USA)	2013	152	15	2.3.1
CVM N51252	Chicken wings	Colorado (USA)	2013	152	15	2.3.1
CVM N51981	Chicken breast	Louisiana (USA)	2013	152	15	2.3.1
CVM N43447	Chicken breast	California (USA)	2013	152	15	2.3.1
13562	Chicken breast	Iowa (USA)	2001	152	15	2.3.1
CVM N43450	Chicken breast	California (USA)	2013	152	15	2.3.2
CVM N43478	Chicken breast	Oregon (USA)	2013	152	15	2.3.2
CVM N43820	Chicken breast	Georgia (USA)	2013	152	15	2.3.2
SA20030505	Chicken cecal contents	Ontario (Canada)	2002	152	15	2.3.2
ABBSB1008-2	Chicken feces	British Columbia (Canada)	2006	152	15	2.3.2
CVM N45412	Chicken breast	Washington (USA)	2013	152	15	2.3.3.1
CVM N47718	Chicken breast	New Mexico (USA)	2013	152	15	2.3.3.1
CVM N47730	Chicken breast	New Mexico (USA)	2013	152	15	2.3.3.1
CVM N51241	Chicken breast	California (USA)	2013	152	15	2.3.3.1
CVM N47729	Chicken breast	Washington (USA)	2013	unknown	15	2.3.3.1
CVM N45934	Chicken breast	Maryland (USA)	2013	152	15	2.3.3.2
CVM N45937	Chicken breast	Maryland (USA)	2013	152	15	2.3.3.2
CVM N45939	Chicken breast	Maryland (USA)	2013	152	15	2.3.3.2
CVM N45944	Chicken breast	Maryland (USA)	2013	152	15	2.3.3.2
CVM N46849	Chicken breast	Pennsylvania (USA)	2013	152	15	2.3.3.2
CVM N47721	Chicken breast	New Mexico (USA)	2013	152	15	2.3.3.2
CVM N47723	Ground turkey	New Mexico (USA)	2013	152	15	2.3.3.2
CVM N48688	Chicken breast	Maryland (USA)	2013	152	15	2.3.3.2
CVM N48710	Chicken breast	Tennessee (USA)	2013	152	15	2.3.3.2
CVM N47722	Ground turkey	New Mexico (USA)	2013	152	15	2.3.3.2
CVM N48687	Chicken breast	Maryland (USA)	2013	152	15	2.3.3.2
CVM N48705	Chicken breast	Louisiana (USA)	2013	152	15	2.3.3.2
CVM N44693	Chicken wings	California (USA)	2013	152	15	2.3.3.2
CVM N43448	Chicken wings	California (USA)	2013	152	15	2.4.1.1
CVM N43455	Chicken breast	Colorado (USA)	2013	152	15	2.4.1.1
CVM N43835	Chicken breast	Washington (USA)	2013	152	15	2.4.1.1
CVM N46820	Chicken breast	Colorado (USA)	2013	152	15	2.4.1.1
CVM N46857	Chicken breast	Pennsylvania (USA)	2013	152	15	2.4.1.1
CVM N50419	Chicken breast	Connecticut (USA)	2013	152	15	2.4.1.1
CVM N50421	Chicken wings	Georgia (USA)	2013	152	15	2.4.1.1
CVM N51294	Chicken breast	New Mexico (USA)	2013	152	15	2.4.1.1
CVM N51313	Chicken breast	Tennessee (USA)	2013	152	15	2.4.1.1
ABB07-SB3057-2	Chicken feces	British Columbia (Canada)	2005	152	15	2.4.1.1
ABB1087-1	Chicken feces	British Columbia (Canada)	2005	152	15	2.4.1.1
22694	Chicken breast	Massachusetts (USA)	2002	152	15	2.4.1.1
CVM N51982	Chicken wings	Louisiana (USA)	2013	152	15	2.4.1.1
CVM29188	Chicken breast	Georgia (USA)	2003	152	15	2.4.1.1
CVM N43465	Chicken breast	Minnesota (USA)	2013	152	15	2.4.1.2
CVM N43466	Chicken wings	Minnesota (USA)	2013	152	15	2.4.1.2
CVM N44708	Chicken breast	New York (USA)	2013	152	15	2.4.1.2
CVM N47720	Chicken breast	New Mexico (USA)	2013	152	15	2.4.1.2
CVM N48707	Chicken breast	New York (USA)	2013	152	15	2.4.1.2
CVM N48711	Chicken breast	Tennessee (USA)	2013	152	15	2.4.1.2
CVM N50437	Chicken wings	New York (USA)	2013	152	15	2.4.1.2
CVM N51256	Chicken wings	Connecticut (USA)	2013	152	15	2.4.1.2
CVM N51273	Chicken breast	Maryland (USA)	2013	152	15	2.4.1.2
ARS-CC5795	Poultry	Maryland (USA)	2010	152	15	2.4.1.2
ARS-CC5805	Poultry	Maryland (USA)	2010	152	15	2.4.1.2
CVM N50429	Chicken breast	New Mexico (USA)	2013	152	15	2.4.1.2
CVM N51277	Chicken breast	Maryland (USA)	2013	152	15	2.4.1.2
CVM N51249	Chicken breast	Colorado (USA)	2013	2132	15	2.4.1.2
CVM N43471	Chicken breast	New Mexico (USA)	2013	152	15	2.4.1.3
SALC-205-3	Chicken feces	British Columbia (Canada)	2004	152	15	2.4.2
20793	Chicken breast	Georgia (USA)	2002	152	15	2.4.2
N312	Chicken breast	Minnesota (USA)	2004	152	15	2.4.2
ARS-CC938	Milk filter (dairy cow farm)	Florida (USA)	2003	198	56	198.1
ATCC 9263	unknown	unknown	Unknown	198	56	198.1
CVM N51290	Ground beef	New Mexico (USA)	2013	198	56	198.1
ARS-CC273	Dairy calf feces	Florida (USA)	2011	198	56	198.1
ARS-CC274	Dairy calf feces	Florida (USA)	2011	198	56	198.1
CVM N41913	Ground beef	Minnesota (USA)	2012	198	56	198.1
CVM N42453	Ground turkey	California (USA)	2012	unknown	56	198.1
915c	Human clinical (sacral wound)	Kuwait	2012	198	56	198.2
917c	Human clinical (stool)	Kuwait	2012	198	56	198.2
CVM 43824	Human clinical	unknown	2012	198	56	198.2
CVM 43780	Human clinical	unknown	2011	198	56	198.2
CVM 43756	Human clinical	unknown	2012	198	56	198.2

^1^ as determined by the Center for Genomic Epidemiology web server [37].

^2^ determined by the University of Warwick MLST database [6]. ST complex assignment was given to unknown ST based on their similarity to known ST.

^3^ based on MP tree inferred from SNPs identified by Parsnp (See Fig 2).

For in-house isolates (bovine-associated isolates and two poultry isolates, and ATCC 9263) antibiotic susceptibility tests (AST) were conducted. All bovine-associated isolates and ATCC 9263 were susceptible to all tested antibiotics. Poultry isolate ARS-CC5795 was resistant to amoxicillin/clauvulanic acid, ampicillin, cefitoxin, ceftiofur, and ceftriaxone, while ARS-CC5805 (poultry isolate) was resistant to streptomycin and tetracycline. The absence of antibiotic resistance in a diverse collection of *S*. Kentucky recovered from dairy cows is consistent with other studies and is notable due to the reported prevalence of antibiotic resistance among poultry-associated *S*. Kentucky within the United States [4][10][11][12][13][42][43]. Antibiotic resistance conferring plasmids are frequently identified in poultry-associated *S*. Kentucky ST152 isolates [12] while antibiotic resistance in human clinical S. Kentucky ST198 is associated with acquisition of the *Salmonella* Genomic Island 1 (SGI1), plasmids, and core genome polymorphisms [7][9][44][45].

### Phylogenetic Inference

To date at least 15 *S*. Kentucky sequence types (ST) have been described MLST studies [7][46]. In these assays, each ST differs from another ST by at least one of seven alleles with some sequence types of the same serovar sharing no alleles. Based on the number of deposited sequences in the MLST database, ST152 and ST198 are among the most frequently isolated *S*. Kentucky sequence types. However, they share no common alleles indicating they are highly diverged from each other [7]. These two STs are part of two larger groups of “ST complexes” which consist of closely related STs. ST152 is a member of the ST15 complex which to date also includes ST151, 212, 318, and 723 among several others. ST198 is a member of ST56 complex, which also includes ST727, 835, and 1680. Outside of these defined complexes there are other closely related STs sharing one or more alleles with members of each complex. A pairwise comparison of seven MLST loci of ST152 and ST198 identified 42 SNPs resulting in a 1.25% sequence divergence (data not shown). When applying the same MLST criteria to the *S*. Kentucky genomes used in this study, three STs were identified (Table 1). All of the poultry-associated isolates were ST152 with one identified as 2132 (one allele difference with ST152), while two bovine-associated isolates were ST318 (one allele difference with ST152 and ST2132), 42 were ST152, and three were ST198. Two strains could not be accurately typed due to abbreviated genes at the ends of contigs. *S*. Kentucky CVM N47729, isolated from a chicken breast, shared six of seven alleles with ST152 and *S*. Kentucky CVM N42453 shared six of seven alleles with ST198. Eight *S*. Kentucky genomes gathered from the NCBI database were identified as ST198. These included five human clinical isolates, two ground beef isolates, and the *S*. Kentucky type strain ATCC 9263. These results are consistent with those of the MLST database in that ST152 from the United States are commonly isolated from poultry and cattle. A single ST318 isolate is included in the MLST database and, consistent with our study, was isolated from cattle in the mid-Atlantic region of the United States. Based on data entries in the MLST database ST198 isolates were recovered on an apparently broader global scale and were more frequently associated with human infections and poultry, cattle, and occasionally other organisms, and the environment.

**Fig 1:**
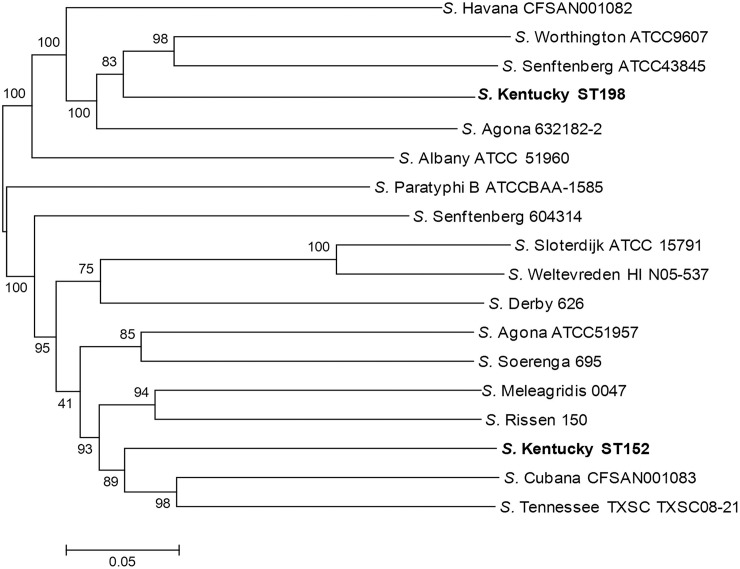
Phylogenetic relationships of *S*. Kentucky ST152 and ST198 with representatives of subclade A1 serovars as described by Timme et al. [16] inferred using the Maximum Likelihood method with the General Time Reversible model of nucleotide substitution. Bar length represents number of substitutions per site.

To investigate the relatedness among the *S*. Kentucky isolates and infer their evolutionary history on a genome-wide scale, SNPs across the genomes of all *S*. Kentucky and closely related serovars were identified. Using a core-genome SNP matrix determined from representative genomes of *S*. *enterica* subclade A1 as described by Timme et al. [16], two distant lineages of *S*. Kentucky were identified (Fig 1). These two *S*. Kentucky lineages correspond to the ST15 complex (ST152, 318 and 2132) and ST56 complex (ST198) of *S*. Kentucky sequence types described above (Table 1).

**Fig 2:**
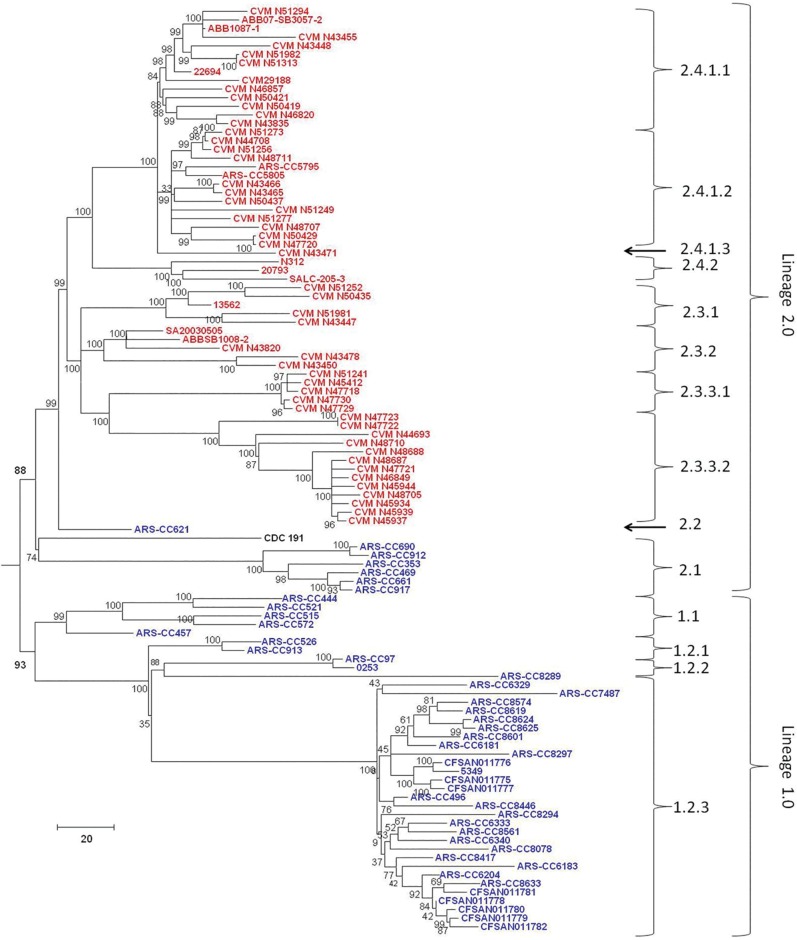
Maximum Parsimony tree of *S*. Kentucky ST152/318/2132 genomes based on 40,795 SNPs detected by Parsnp and rooted in twelve *S*. Kentucky ST198 isolates. Bovine-associated strains are labeled in blue, poultry-associated strains are labeled in red, and the human clinical isolate CDC 191 is labeled in black. Clusters of genomes are labeled on right of tree. Tree was inferred with 1000 bootstrap replicates. Bar length = number of substitutions.

**Fig 3:**
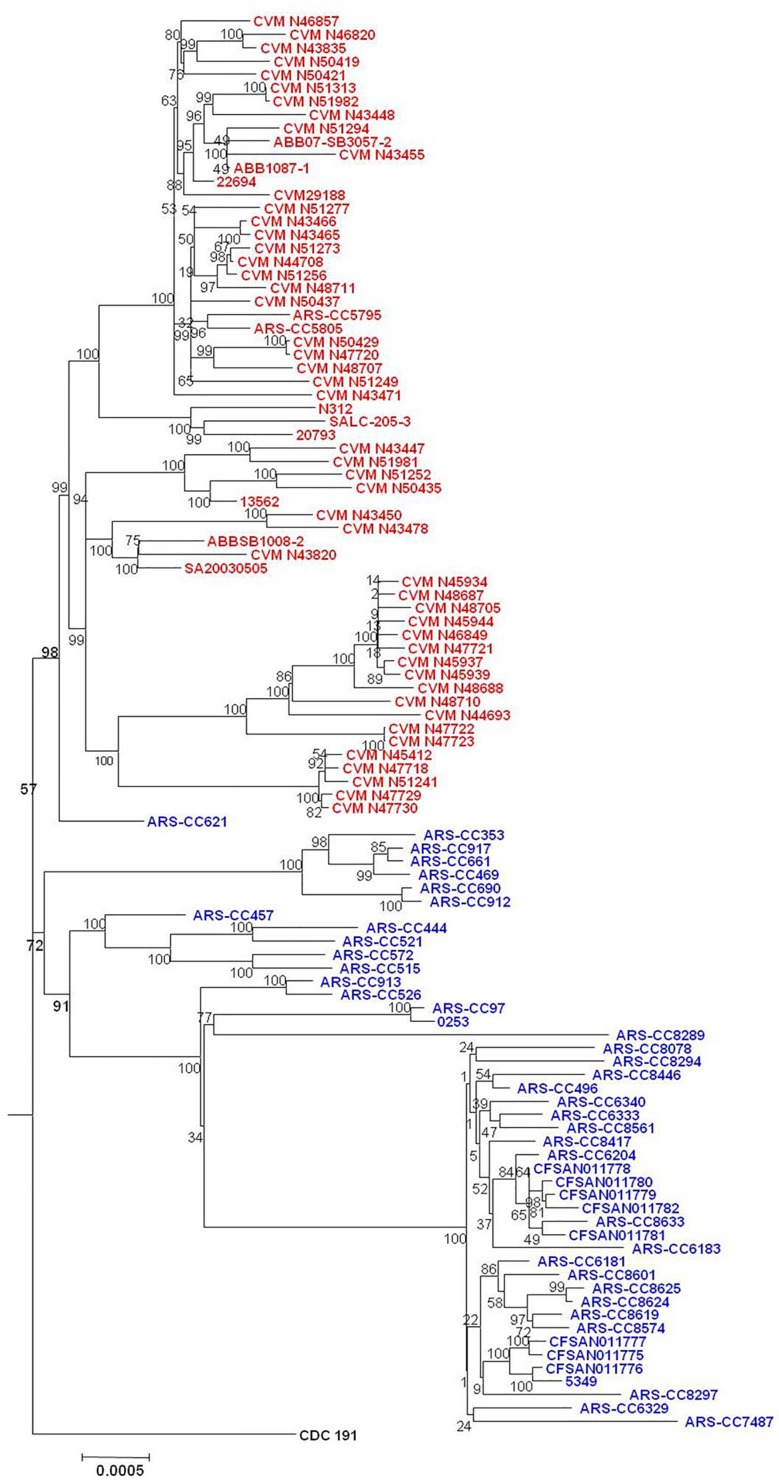
Maximum Likelihood tree of *S*. Kentucky ST152/318/2132 genomes based on SNPs detected by Parsnp. Bovine-associated strains are labeled in blue, poultry-associated strains are labeled in red, and the human clinical isolate CDC 191 is labeled in black. Tree was inferred using the General Time Reversible model of nucleotide substitution in RAxML with 1000 bootstrap replicates and rooted in twelve *S*. Kentucky ST198 genomes. Bar = number of substitutions per site.

**Fig 4:**
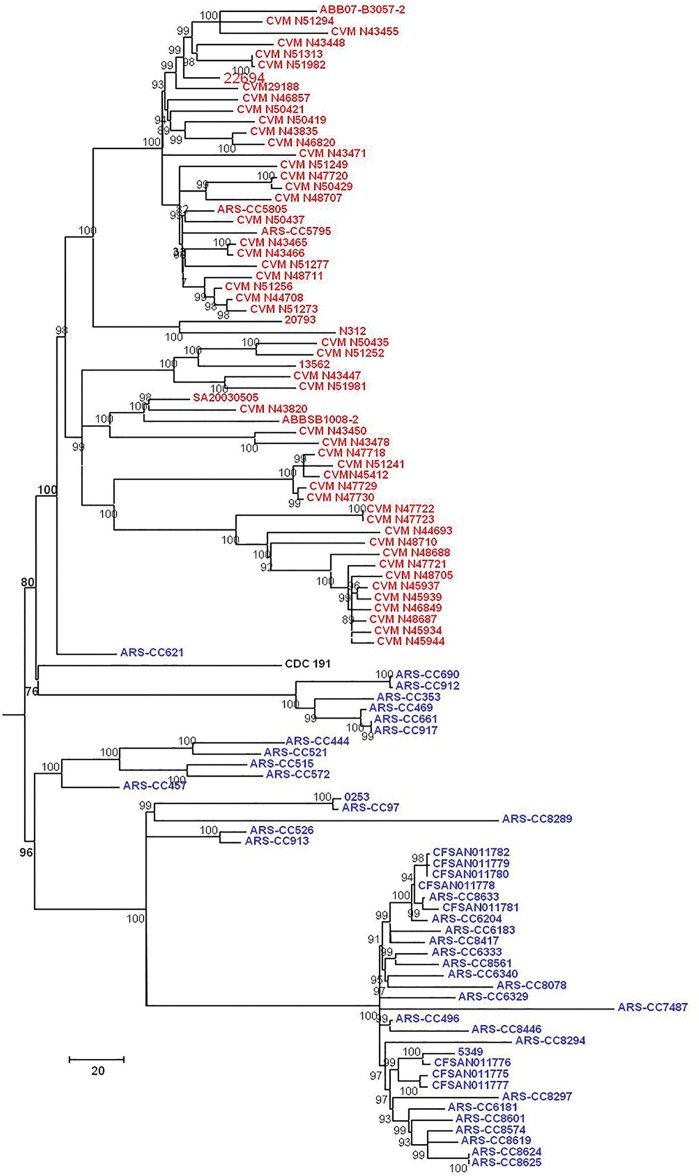
Maximum Parsimony tree of *S*. Kentucky ST152/318/2132 genomes based on 45,999 SNPs detected by Lyve-SET and rooted in twelve *S*. Kentucky ST198 genomes. Bovine-associated strains are labeled in blue, poultry-associated strains are labeled in red, and the human clinical isolate CDC 191 is labeled in black. Tree was inferred with 1000 bootstrap replicates. Bar length = number of substitutions.

**Fig 5:**
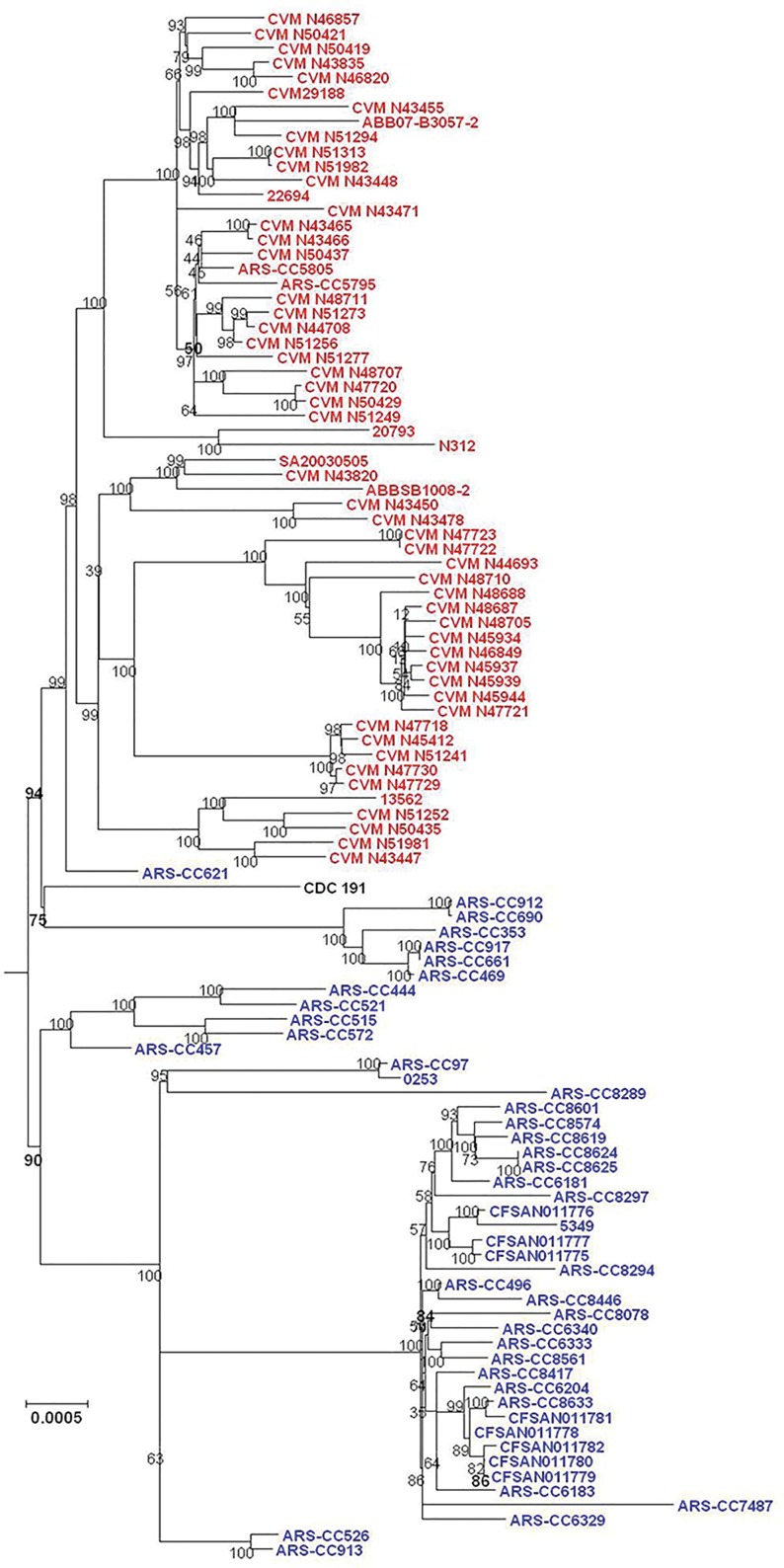
Maximum Likelihood tree of *S*. Kentucky ST152/318/2132 genomes based on SNPs detected by Lyve-SET. Bovine-associated strains are labeled in blue, poultry-associated strains are labeled in red, and the human clinical isolate CDC 191 is labeled in black. Tree was inferred using the General Time Reversible model of nucleotide substitution in RAxML with 1000 bootstrap replicates and rooted in twelve *S*. Kentucky ST198 genomes. Bar = number of substitutions per site.

**Fig 6:**
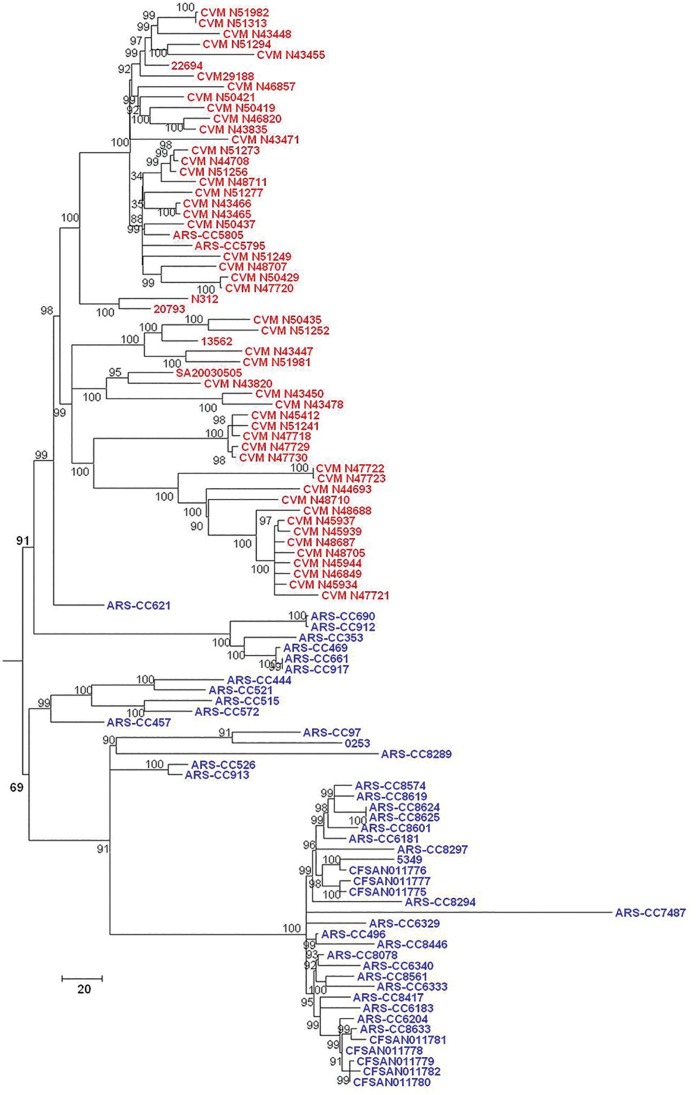
Maximum Parsimony tree of *S*. Kentucky ST152/318/2132 genomes based on 48,269 SNPs detected by CFSAN SNP Pipeline and rooted in seven *S*. Kentucky ST198 isolates. Bovine-associated strains are labeled in blue and poultry-associated strains are labeled in red. Tree was inferred with 1000 bootstrap replicates. Bar length = number of substitutions.

**Fig 7:**
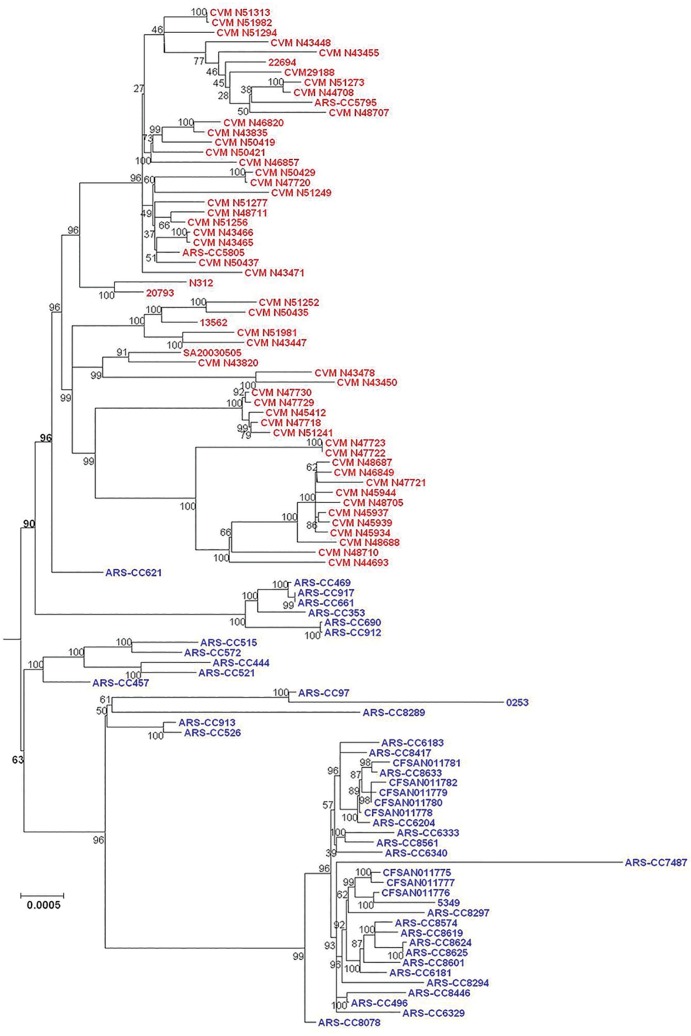
Maximum Likelihood tree of *S*. Kentucky ST152/318/2132 genomes based on SNPs detected by CFSAN SNP Pipeline. Bovine-associated strains are labeled in blue and poultry-associated strains are labeled in red. Tree was inferred using the General Time Reversible model of nucleotide substitution in RAxML with 1000 bootstrap replicates and rooted in seven *S*. Kentucky ST198 genomes. Bar = number of substitutions per site.

SNPs within the *S*. Kentucky genomes (excluding other subclade A1 serovars) were identified using three methods and phylogenetic trees were inferred from these data. Phylogenies inferred from data derived from the three SNP-detection methods were, for the most part, approximately similar in topology. For all SNP detection methods and both methods of tree inference the ST15 complex was observed to have two major sublineages (labeled as Lineages 1.0 and 2.0 in this analysis) (Figs 2–7). For both MP and ML analyses there was weak support for the placement of some sublineages in the ancestral node of the Lineage 1.0 and for the Parsnp ML analysis the human clinical isolate (*S*. Kentucky CDC 191) is rooted in the ancestral node of the tree while it is placed in Lineage 2.0 in the other phylogenetic analyses of this study (Figs 2, 4, and 5).

For five of the six analyses Lineage 1.0 is composed to several clusters of bovine isolates (n = 39) labeled as clusters 1.1, 1.2.1, 1.2.2 and 1.2.3. Cluster 1.1 consists of five ST152 isolates, one recovered from Texas and four from Wisconsin in 2007. The Wisconsin isolates grouped together, separately from the single Texas isolate. Cluster 1.2.1 consisted of one isolate from Pennsylvania and one from Virginia. Cluster 1.2.2 consisted of three isolates, two of them were typed as ST318 and collected from the same farm in Pennsylvania while the third isolate was a highly diverged ST152 isolate collected from Ohio. Cluster 1.2.3 consisted of 29 isolates, mainly from Pennsylvania with one isolate from New York, and two from Ohio. These results, however, are not consistent across all tree inference methods as the ML analysis of the SNPs detected with Parsnp place all bovine isolates except ARS-CC621 in Lineage 1.0 (Fig 3), albeit with low bootstrap support, and consistent with other methods place all poultry isolates in Lineage 2.0.

The topology of Lineage 2.0 is moderately to strongly supported for all analyses and is composed of three to four clusters of strains. For the Parsnp MP and Lyve-SET and CFSAN SNP Pipeline MP and ML analyses the ancestral Lineage 2.0 node moderately to strongly supported (80 to 94%) with much stronger support for the node from which the bovine-associated isolate ARS-CC621 and all poultry-associated isolates descended for all analyses.

For all analyses excluding the Parsnp ML analysis the Lineage 2.0 clusters included one group of bovine/human-associated genomes, (cluster 2.1), one bovine-associated genome (cluster 2.2) and two groups of poultry-associated genomes (clusters 2.3.1, 2.3.2, 2.3.3.1, 2.3.3.2 and 2.4.1.1, 2.4.1.2, 2.4.1.3, and 2.4.2) which is consistent with the two dominant poultry-associated pulsotypes described by Ladely et al. [13]. Cluster 2.1 consists of seven isolates; three from Vermont, one from New York two from Wisconsin, and a distantly related human clinical isolate (CDC 191) from Wisconsin in 2002. Within this group the Vermont isolates are more closely related to each other than they are to the New York and Wisconsin isolates, and the Wisconsin isolates are more closely related to each other than they are to the New York or Vermont isolates. Cluster 2.2 consists of a single bovine-associated isolate (ARS-CC621) recovered in Texas in 2007.

Clusters 2.3.1 to 2.4.2 consist solely of isolates recovered from poultry, poultry products or processing plants and chicken feces/cecum samples collected across North America over a 12 year period. These clusters are monophyletic (geneaological sorting index = 1, P <0.0001) and do not include any bovine isolates indicating complete lineage sorting from the most recent common ancestor that gave rise to poultry-associated isolates. All poultry-associated clusters were moderately to strongly supported for all MP analyses, while ML analyses resulted in lower bootstrap support for clusters 2.4.1.1 to 2.4.2. Within the latter tree, the topologies of cluster 2.4.1 and 2.4.2 are not consistent with those of all other MP and ML trees.

Within the paraphyletic bovine-associated groups presence of different clusters in the same region (Wisconsin, Texas, and Pennsylvania) indicates multiple evolutionary sublineages of *S*. Kentucky ST152 are circulating in dairy cow herds of the same region. For example, clusters 1.1 and 2.1 isolates were recovered from dairy cows in Wisconsin, cluster 1.1 and 2.2 isolates were recovered from Texas, and clusters 1.2.1, 1.2.2 and 1.2.3 isolates were detected in Pennsylvania. Thus, broad geographic dispersal of *S*. Kentucky strains may have occurred repeatedly over time. Dairy farms are open environments in that there is a potential of wildlife intrusions on the farm and interactions with dairy cows [47]. For example, migratory birds are known vehicles of enteric bacteria and have been suggested to be a transmission route to livestock [48] and some wild bird species such as European starlings (*Sturnus vulgaris*) are known to concentrate in areas of cattle operations where their presence has been associated with increased *S*. *enterica* contamination of feed and water [49].

Within the poultry-associated clusters, a clear geographic pattern of distribution was not observed. For example, isolates from New Mexico, Tennessee, California, and British Columbia were identified in both clusters 2.3 and 2.4. Along with the presence of isolates from the same state in multiple clusters, geographically disparate isolates grouped together as well. For instance, some isolates from British Columbia (ABBSB1008-2) are sister taxa on the same phylogenetic sublineage with one from Georgia (CVM N43820), two regions that are separated by over 4500 km. Similarly, isolates from California also clustered with those from Tennessee, Massachusetts, New York, Colorado, and Washington State in cluster 2.4. This lack of geographic clustering among the poultry isolates is most likely due to the fact that the majority of these isolates were not collected on-farm, but rather after poultry product processing. Sequencing of isolates collected from farms would help identify any possible geographic signatures present in the currently circulating ST152 populations.

Based on the genomes analyzed in this study there is strong evidence to support the hypothesis that bovine-associated ST152 isolates are phylogenetically distinct from poultry-associated ST152 isolates. However, these data only reflect those *S*. Kentucky ST152 isolates collected in North America in recent years. It is known that ST152 isolates have been recovered from various sources worldwide and as far back as the 1950s, but what is not well documented in the literature is how frequently ST152 are recovered from cattle and poultry in other continents, or if this ST152-Bovine/Poultry relationship occurs in other regions globally, i.e, if the ST152 strains have entered bovine and/or poultry populations outside of North America or if they are circulating through the populations of other animals. At present, the metadata of only five ST152 strains from outside of North America are deposited in the MLST database and none of these were recovered from poultry or bovine sources, but rather fish meal and human clinical cases. Addition of other ST152 genomes from a variety of sources may result in a somewhat different phylogenetic relationship among strains than what is presented here, as well as the presence of other evolutionary lineages not detected in this study.

### Genomic Polymorphisms within the ST152/ST318/2132 Isolates (ST complex 15)

In total there were 2662 SNPs identified in the core genome analysis by Parsnp, and 3353 and 3336 identified by Lyve-SET and CFSAN SNP Pipeline, respectively. We further identified Lineage and host-associated SNPs. The Lineage 1.0/2.0 divergence event for all trees excluding the Parsnp ML tree is marked by eight identified SNPs; four in intergenic regions and four in protein coding genes resulting in three synonymous mutations and one non-synonymous mutation (SeKA_A1002, SeKA_A1027, SeKA_A1948, SeKA_A4700) (Table 2).

**Table 2:** Identified SNPs defining the Lineage 1.0/2.0 divergence (Top) and the Bovine-associated/Poultry-associated divergence (Bottom). S = synonymous mutation. NS = non-synonymous mutation.

Lineage 1.0 / 2.0	Locus Tag ^a^	Annotation ^b^	Genome Position ^a^	nt Lineage 2.0	nt Lineage 1.0	Substitution ^c^	aa Lineage 2.0	aa Lineage 1.0	aa Position ^a^	Method
	intergenic		528068	C	T					1, 2, 3
	SeKA_A1002	LysR substrate binding domain protein	953413	A	G	S	Leu	Leu	223	1, 2, 3
	SeKA_A1027	nitrite extrusion protein 2	984718	C	T	S	Pro	Pro	288	1, 2, 3
	intergenic		1032798	A	G					2, 3
	SeKA_A1948	amidophosphoribosyltransferase	1876321	G	A	S	Ala	Ala	288	1, 2, 3
	intergenic		2432705	T	G					1, 2, 3
	intergenic		3169966	T	G					1, 2, 3
	SeKA_A4700	propionate—CoA ligase	4587482	A	C	NS	Asn	Thr	240	2, 3
Bovine / Poultry	Locus Tag ^a^	Annotation ^b^	Genome Position ^a^	nt Poultry-associated	nt Bovine-associated	Substitution ^c^	aa Poultry	aa Bovine	aa Position	Method
	SeKA_A1094	protein YdcF	1055798	T	C	NS	Thr	Ala	43	1, 2, 3
	SeKA_A2591	hemolysin-3	2519134	A	C	NS	Phe	Val	32	3
	SeKA_A2812	methyltransferase family protein	2713142	G	A	NS	Ser	Leu	39	1, 2, 3
	SeKA_A4467	carbonate dehydratase	4347265	G	T	NS	Ala	Asp	22	1, 2, 3

^a^ = Locus/Position in *S*. Kentucky CVM29188

^b^ = Annotation in *S*. Kentucky CVM29188 (accession no. ABAK02000001.1)

^c^ = S(synonymous), NS(nonsynonymous)

1 = Parsnp

2 = Lyve-SET

3 = CFSAN SNP Pipeline

In a comparison of bovine-associated isolates to poultry-associated isolates an average of 217, 247, and 281 SNP differences were identified between the two by the Parsnp, Lyve-SET, and CFSAN SNP Finder analyses, respectively (Parsnp range = 62 to 308 SNPs, Lyve-SET range = 72 to 377 SNPs, and CFSAN SNP Pipeline range = 72 to 489). Within these SNP matrices there were four SNPs in protein coding genes, all resulting in non-synonymous mutations, that defined the bovine-poultry divergence for all strains (SeKA_A1094, SeKA_A2591, SeKA_A2812, SeKA_A4467) (Table 2). No evidence of the role of these genes in host-specificity, colonization of the gut by *S*. *enterica* or persistence of *S*. *enterica* in cows, poultry, poultry and bovine products, or the environment could be found in the literature. However, hemolysin-3 (SeKA_A2591) has been shown in Gram-negative and Gram-positive organisms to be involved in the infection process of other mammals, particularly *Vibrio vulnificus* and *Bacillus cereus* [50][51]. The hemolysin-3 in *S*. Kentucky demonstrates 71% and 45% amino acid identity to that of *V*. *vulnificus* and *B*. *cereus*. *In vitro* and *in vivo* assays need to be conducted to further elucidate the potential roles of these protein-coding genes in persistence in the poultry and bovine hosts or specificity to either environment.

**Fig 8:**
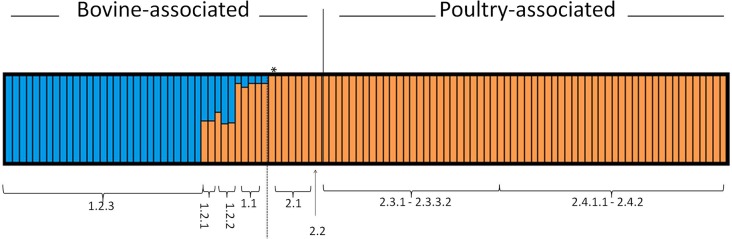
STRUCTURE analysis of the proportion of each isolate’s SNP profile attributed to each of the different groups. Dashed line = Lineage 1.0/2.0 divergence. Profiles to the left of the solid black line = bovine-associated isolates, while profiles to the right of the solid black line = poultry-associated isolates. Asterisk = human clinical isolate CDC 191.

A Bayesian analysis of SNP characters using the STRUCTURE v 2.3.4 program was consistent with the inferred ML and MP phylogenies (Fig 8) in showing that there were primarily two distinct groups. However, these results provided additional details that help explain the placement of certain isolates within the phylogenetic analysis. For example, STRUCTURE showed that isolates within Cluster 1.2.3 have SNP profiles that are, for the most part, distinct from all others (blue profiles). In contrast, clusters 1.1, 1.2.1, and 1.2.2 showed evidence of admixture (blue/orange profiles) where those isolates had some proportion of SNPs that are indicative of clusters 1.2.3 (blue profiles) and Lineage 2.0 isolates (orange profiles). Cluster 1.1 isolates had SNP profiles more closely related to clusters 2.1 to 2.4 than to 1.2.3. All poultry isolates all show approximately similar SNP profiles with the basal cow isolates in clusters 2.1 and 2.2 indicating a high level of similarity among the cow and poultry isolates within evolutionary Lineage 2.0.

### Plasmid Detection

Using the assembled genomes, multiple plasmids were detected in the *S*. Kentucky ST152 isolates while several were identified as plasmid-free (Table 3 and S2 Table). Thirty three poultry-associated isolates encoded sequences similar to the ColV plasmid pCVM29188_146 plasmid (IncFIB), which is somewhat consistent with previous studies of poultry-isolated *S*. Kentucky in North America that have shown that the majority of these strains (72.9%) harbored markers of ColV plasmids [52]. However, this study also demonstrated that *S*. Kentucky isolates collected on farms were more likely to harbor this plasmid than those collected from retail meats [52], while the majority of isolates analyzed here were recovered from the latter. This plasmid has been shown to be involved in enhanced survival of *S*. Kentucky in poultry and is highly similar in gene content and nucleotide sequence similarity to the ColV plasmid of avian pathogenic *Escherichia coli* (APEC) [52]. The presence of this plasmid was restricted to poultry-associated isolates, providing further evidence that it is involved in interactions with the poultry host. However, it should be noted that Fricke et al. [12] identified six bovine-associated isolates with PCR markers indicative of the presence of APEC-like plasmids similar to those of pCVM29188_146 suggesting that more bovine-specific *S*. Kentucky need to be evaluated for the presence of this or highly similar plasmids. Although, Johnson et al. [52]demonstrated the role of this plasmid in enhanced extracellular survival in poultry, its absence from poultry-associated *S*. Kentucky suggests there are other factors involved in specificity of these strains to the poultry host. Similar to Ladely et al. [13] the IncFIB plasmid type was not evenly distributed across poultry-associated strains as it was identified in 81% of cluster 2.4.1.1 to 2.4.2 isolates but only in 25% of cluster 2.3.1 to 2.3.3.2 isolates.

**Table 3:** Plasmids identified by PlasmidFinder in genomes analyzed in this study.

ST	Poulty/Bovine	Plasmid Replicon	Number of Plasmids Detected
ST152	Poultry-associated	IncFIB	33
		IncX1	58
		IncHI2	12
		Col156	2
		IncI1	29
	Bovine-associated	IncI1	34
		IncI2	3
		IncA/C2	4
		ColpVC	1
		Col8282	3
		No plasmid detected	12
ST198		IncI1	1
		No plasmid detected	11

The most frequently detected plasmid sequences in poultry-associated isolates were those similar to the canonical pCVM29188_46 (IncX1) plasmid, which was also not detected in any of the bovine-associated isolates (Table 3 and S2 Table). The biological role of this plasmid has not been well-elucidated, but its high prevalence among these strains indicates it may play a significant role in the survival of *S*. Kentucky within the poultry host, poultry production environment, or in transmission of *S*. Kentucky between animals. However, this would need to be further evaluated *in vivo*. Coupling the plasmid presence/absence data with the inferred phylogeny suggests that poultry-associated *S*. Kentucky acquired this plasmid, as well as the IncFIB plasmid after diverging from the most recent common ancestor shared with the bovine-associated isolates.

Within the bovine-associated *S*. Kentucky 12 isolates were identified as plasmid-free and these isolates belonged to clusters 1.1, 1.2.1, 2.1, and 2.2 (Table 3 and S2 Table). Isolates from several clusters harbored a variety of plasmids, including those belonging to IncI1 (34 isolates), IncI2 (3 isolates), and IncA/C2 (4 isolates) replicon types, and plasmids identified by PlasmidFinder as Col8282 (3 isolates) and one as ColpVC. The IncI1 plasmid, represented by sequence AOYZ01000068.1 in GenBank of *S*. Kentucky 5349, was the most frequently detected plasmid sequence among the bovine-associated isolates. This sequence is ca. 92 kb, encodes 100 ORFs and is somewhat similar to plasmid sequences available in the NCBI database. These include plasmid pSTY1-1898 of *S*. Typhimurium str. USDA-ARS-USMARC-1898 (coverage = 81%, similarity = 99%) and *S*. Kentucky plasmid pCS0010A_95 (coverage = 79%, similarity = 99%), among several others.

The majority of isolates that harbored plasmids were from the mid-Atlantic region of the United States. The absence of plasmids in many bovine-associated isolates (25%) suggests they are not necessary for survival or persistence within the bovine gastrointestinal tract or the dairy farm environment. However, the high level prevalence of IncI1 plasmids in cluster 1.2.3 isolates indicates that they may provide an advantage in these environments, and their restriction to isolates from the mid-Atlantic and Northeast United States may be indicative of their importance to the ecology of these strains in this region.

### Phylogeny and Genomic Polymorphisms within the ST198 Isolates (ST complex 56)

**Fig 9:**
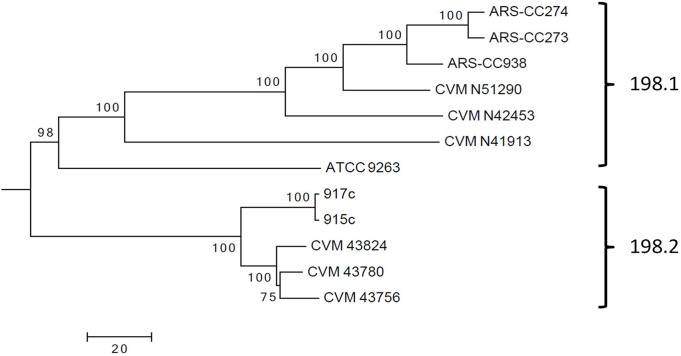
Maximum Parsimony tree of *S*. Kentucky ST198 isolates collected from human clinical cases (cluster 198.2) and dairy cows and food products in North America (198.1). The tree was inferred from an alignment of SNPs detected among all *S*. Kentucky ST198 genomes and using the chromosome of *S*. Kentucky CVM29188 as a reference. The tree is based on 42,958 SNPs (Parsnp analysis), of these, 532 SNPs were identified among the ST198 genomes. The tree displayed here is rooted in the distantly related *S*. Kentucky CVM29188. Tree was inferred with 1000 bootstrap replicates. Bar length = number of substitutions.

Within the *S*. Kentucky ST198 lineage two major clusters were identified (labeled here as clusters 198.1 and 198.2) (Fig 9). Cluster 198.1 consists of isolates recovered from agricultural sources in the United States (dairy cow feces, dairy cow milk, ground beef, and ground turkey) and the *S*. Kentucky type strain ATCC 9263. Cluster 198.2 consists of five human clinical isolates, two of these collected from the same patient in Kuwait in 2012 (915c from a sacral wound and 917c from a stool sample) [25], and three were collected from human clinical cases in the United States. It is not known if these three infections were acquired abroad or within the United States, as metadata for these isolates are lacking in the public database. Source attribution would need to be verified before the locations of infections can be confirmed. *S*. Kentucky ST198 infections have been reported in Africa, Eastern Europe, and Southeast Asia and travel-acquired infections with these organisms has been reported in people traveling to these regions [7][8]. Until a more comprehensive geophylogeny of *S*. Kentucky ST198 strains is conducted assumptions about strain traceback are speculative.

Using Parsnp, with *S*. Kentucky CVM29188 as a reference genome, there were 532 SNPs identified in the core-genomes of the ST198 isolates with an average of 209 SNP differences between the clusters 198.1 and 198.2 genomes (range = 169 to 228 SNPs). Cluster 198.1 isolates collected from agricultural sources in the United States had, for the most part, fewer SNPs between each other (mean = 138, range = 10 to 210) than they did with the human clinical isolates (cluster 198.2) (Table 4). Similarly, the human clinical isolates (cluster 198.2) had fewer SNP differences between each other than they did with the cluster 198.1 isolates (mean = 32, range = 2 to 48). Interestingly, in a genome-genome comparison fewer SNPs were detected between ATCC 9263 and 198.2 genomes than between ATCC 9263 and 198.1 genomes. However, there were fewer SNPs between ATCC 9263 and the 198.1 cluster than ATCC 9263 and the 198.2 cluster when the average base pair differences over all of the sequence pairs were calculated per cluster.

**Table 4:** SNP differences among *S*. Kentucky ST198 genomes.

Cluster / Genome		ATCC 9263	CVM N51290	CVM N42453	CVM N41913	ARS-CC274	ARS-CC273	ARS-CC938	CVM 43780	CVM 43756	CVM 43824	915c
198.1	ATCC 9263											
	CVM N51290	195										
	CVM N42453	201	94									
	CVM N41913	200	193	197								
	ARS-CC274	210	69	107	206							
	ARS-CC273	210	71	111	210	10						
	ARS-CC938	197	58	98	197	35	33					
198.2	CVM 43780	169	204	210	209	219	219	206				
	CVM 43756	178	211	215	214	224	228	215	19			
	CVM 43824	174	207	211	210	220	224	211	17	22		
	915c	177	208	214	213	223	225	212	41	48	44	
	917c	179	210	214	213	223	227	214	43	48	44	2

There were 66 conserved SNP differences identified between clusters 198.1 and 198.2 (Table 5). Of particular interest are the DNA gyrase subunit A (*gyrA*) (AEX15_13770) substitutions Ser83 → Phe in 198.2; Asp87 → Tyr in CVM 43780, CVM 43756, and CVM 43824; and Asp87 → Asn in 915c and 917c. These substitutions confer resistance to nalidixic acid, an attribute that is presumed to have emerged in ST198 isolates in the early 2000s in Egypt[7]. Substitution Ser80 → Ile in DNA topoisomerase IV subunit A (*parC*) (AEX15_04910) conferring resistance to ciprofloxacin, was also observed in 198.2 isolates. These substitutions are characteristic of ST198 strains circulating in North and East Africa and the Middle East [7] suggesting that the infections caused by these strains (*S*. Kentucky CVM 43780, CVM 43756, and CVM 43824) may have been acquired outside of the United States or from exposure to imported products.

**Table 5:** SNP differences between 198.1 (non-clinical North American agricultural isolates) and 198.2 (human clinical isolates). The reference genome for this analysis is the chromosome of *S*. Kentucky CVM29188.

Locus Tag in CVM N51290	Locus Tag in CVM29188	Annotation ^a^	Genome Position ^a^	nt 198.1	nt 198.2	Substitution ^b^	aa 198.1	aa 198.2	aa Position
AEX15_00985	SeKA_A0118	putative cytoplasmic protein	112694	A	G	NS	Asn	Ser	215
AEX15_01465	SeKA_A0272	ABC transporter ATP-binding protein	259861	G	A	S	Asn	Asn	352
AEX15_07125	SeKA_A0450	outer membrane protein F	449431	G	A	S	Ala	Ala	310
intergenic			511489	G	A				
intergenic			528058	C	G				
intergenic			768380	G	T				
intergenic			932324	T	C				
AEX15_09820	SeKA_A1028	Nitrate reductase alpha subunit	986092	C	A	NS	Ser	Tyr	249
AEX15_09905	SeKA_A1046	virulence factor SrfB	1006926	C	T	S	Asn	Asn	389
AEX15_10005	SeKA_A1065	PTS system, IIc component	1028557	T	G	NS	Val	Gly	232
AEX15_10580	SeKA_A1172	phosphatidylglycerophosphatase B	1132142	T	C	NS	Thr	Ala	184
AEX15_10645	SeKA_A1188	anthranilate synthase component I	1148000	C	T	NS	Pro	Ser	225
AEX15_11230	SeKA_A1320	hypothetical protein	1268383	G	C	NS	Gly	Ala	47
AEX15_11825	SeKA_A1435	aspartyl-tRNA synthetase	1376156	C	T	NS	Ala	Thr	273
AEX15_11880	SeKA_A1448	flagellar protein FlhE	1388508	T	C	S	Leu	Leu	624
AEX15_11945	SeKA_A1461	transcriptional activator FlhD	1402136	G	T	S	Ser	Ser	4
AEX15_12480	SeKA_A1563	arsenical pump-driving ATPase	1479380	A	G	NS	Leu	Ser	578
AEX15_13315	SeKA_A1750	beta-D-glucoside glucohydrolase	1662011	G	A	S	Thr	Thr	560
AEX15_13615	SeKA_A1814	nucleoid-associated protein NdpA	1730145	T	C	NS	Asn	Asp	136
AEX15_13770	SeKA_A1846	DNA gyrase subunit A (*gyrA*)	1764089	G	A	NS	Ser	Phe	83
AEX15_13940	SeKA_A1884	O-succinylbenzoate synthase	1805065	C	A	NS	Glu	Asp	44
AEX15_15785	SeKA_A2190	DNA repair protein RecO	2129001	T	C	NS	Lys	Glu	156
AEX15_21540	SeKA_A2229	phospho-2-dehydro-3-deoxyheptonate aldolase	2169354	G	A	S	Asn	Asn	149
AEX15_21995	SeKA_A2343	murein hydrolase effector LrgB	2282024	G	A	S	Gly	Gly	38
AEX15_22185	SeKA_A2383	putative type III secretion system effector protein OrgC	2317497	G	A	NS	Ser	Leu	98
AEX15_22530	SeKA_A2451	sulfate adenylyltransferase subunit 1	2379190	A	C	NS	Ile	Ser	339
intergenic			2476378	G	A				
AEX15_04160	SeKA_A2564	nickel/cobalt efflux protein RcnA	2496525	C	T	NS	Pro	Ser	31
intergenic			2497451	C	G				
AEX15_04380	SeKA_A2619	D-erythrose-4-phosphate dehydrogenase	2542815	G	A	NS	Pro	Ser	224
AEX15_04910	SeKA_A2749	DNA topoisomerase IV subunit A (*parC*)	2654521	C	A	NS	Ser	Ile	80
intergenic			2760667	C	A				
AEX15_03530	SeKA_A3124	cytoplasmic protein	2998220	G	A	S	Thr	Thr	395
AEX15_02625	SeKA_A3321	putative glucarate transporter	3208206	T	A	NS	Leu	Gln	355
AEX15_02555	SeKA_A3336	ADP-heptose—LPS heptosyltransferase	3221877	G	A	S	Pro	Pro	122
AEX15_02175	SeKA_A3395	PTS sorbose transporter subunit IIC	3280995	C	T	NS	Gly	Asp	241
AEX15_14010	SeKA_A3451	trimethylamine N-oxide reductase I catalytic subunit	3334071	G	A	S	Cys	Cys	320
AEX15_14230	SeKA_A3482	phosphate transporter permease subunit PstC	3369116	G	T	NS	Pro	His	74
AEX15_14255	SeKA_A3487	glucosamine—fructose-6-phosphate aminotransferase	3375283	T	C	NS	Ile	Val	490
AEX15_20905	SeKA_A3529	threonine dehydratase	3421216	G	A	S	Ala	Ala	446
AEX15_21030	SeKA_A3553	putative common antigen polymerase	3445490	G	A	S	Leu	Leu	125
AEX15_21080	SeKA_A3565	porphobilinogen deaminase	3453638	G	T	NS	Asp	Glu	117
AEX15_21220	SeKA_A3595	chondroitin sulfate/heparin utilization regulation protein	3480727	C	T	S	Asn	Asn	103
AEX15_21260	SeKA_A3603	sec-independent translocase	3489186	C	T	NS	Pro	Ser	142
AEX15_21305	SeKA_A3612	proline dipeptidase	3500226	C	T	S	Ser	Ser	179
intergenic			3575800	C	A				
AEX15_21420	SeKA_A3805	thiamine-phosphate pyrophosphorylase	3686081	C	A	NS	Ala	Ser	57
AEX15_18585	SeKA_A3887	LexA repressor	3764969	C	T	S	Ser	Ser	60
intergenic			3770533	C	A				
AEX15_18425	SeKA_A3921	Na+/H+ antiporter	3813232	G	A	S	Ser	Ser	30
intergenic			3829881	C	T				
intergenic			3832063	T	A				
intergenic			3896075	C	T				
AEX15_17850	SeKA_A4040	putative cytoplasmic protein	3929069	T	C	S	Thr	Thr	54
intergenic			4002925	G	A				
AEX15_20245	SeKA_A4293	chitinase	4165915	A	G	S	Val	Val	533
AEX15_20105	SeKA_A4321	isoleucyl-tRNA synthetase	4192511	C	A	S	Thr	Thr	62
AEX15_17125	SeKA_A4346	carnitine operon protein CaiE	4219742	C	T	NS	Glu	Lys	128
intergenic			4233585	C	A				
AEX15_16055	SeKA_A4409	UDP-N-acetylmuramoyl-L-alanyl-D-glutamate synthetase	4286504	T	C	NS	Ser	Pro	265
AEX15_16220	SeKA_A4445	hypothetical protein	4325107	A	G	S	Gly	Gly	137
AEX15_16390	SeKA_A4481	putative fimbrial outer membrane usher protein	4359640	G	T	NS	Leu	Met	704
intergenic			4463447	T	C				
AEX15_22970	SeKA_A4665	fimbrial outer membrane usher protein StbC	4548312	A	C	NS	Val	Gly	119
AEX15_06235	SeKA_A4762	2-aminoethylphosphonate transport system permease PhnU	4645904	A	G	NS	Ile	Thr	92
AEX15_06585	SeKA_A4840	copper exporting ATPase	4725203	A	G	S	Ser	Ser	111

^a^ = Position/annotation in *S*. Kentucky CVM29188 (accession no. ABAK02000001.1)

^b^ = S(synonymous), NS(nonsynonymous)

**Fig 10:**
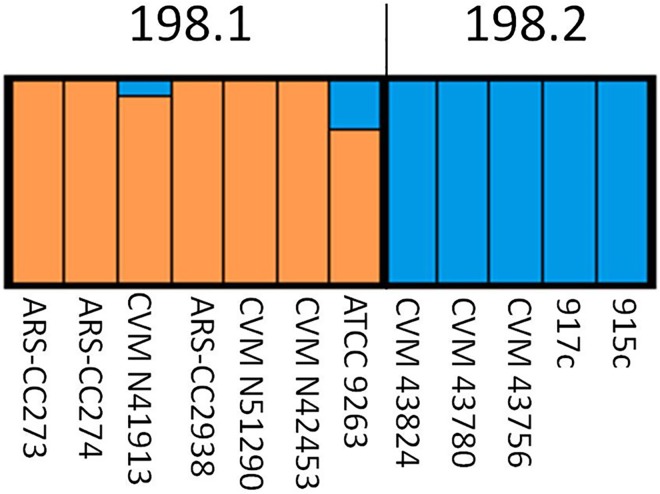
STRUCTURE analysis of the proportion of each isolate’s SNP profile attributed to each of the different clusters (198.1 and 198.2).

A Bayesian analysis of SNP differences among isolates using the STRUCTURE program indicated that for the most part 198.1 and 198.2 isolates were distinct with the exception of ATCC 9263 and CVM N41913, which were basal within the cluster 198.1 lineage (Fig 10). For these two isolates a proportion of SNPs detected in their genomes (ATCC 9263 = 0.24 and CVM N41913 = 0.078) were characteristic of those identified in 198.2.

**Fig 11:**
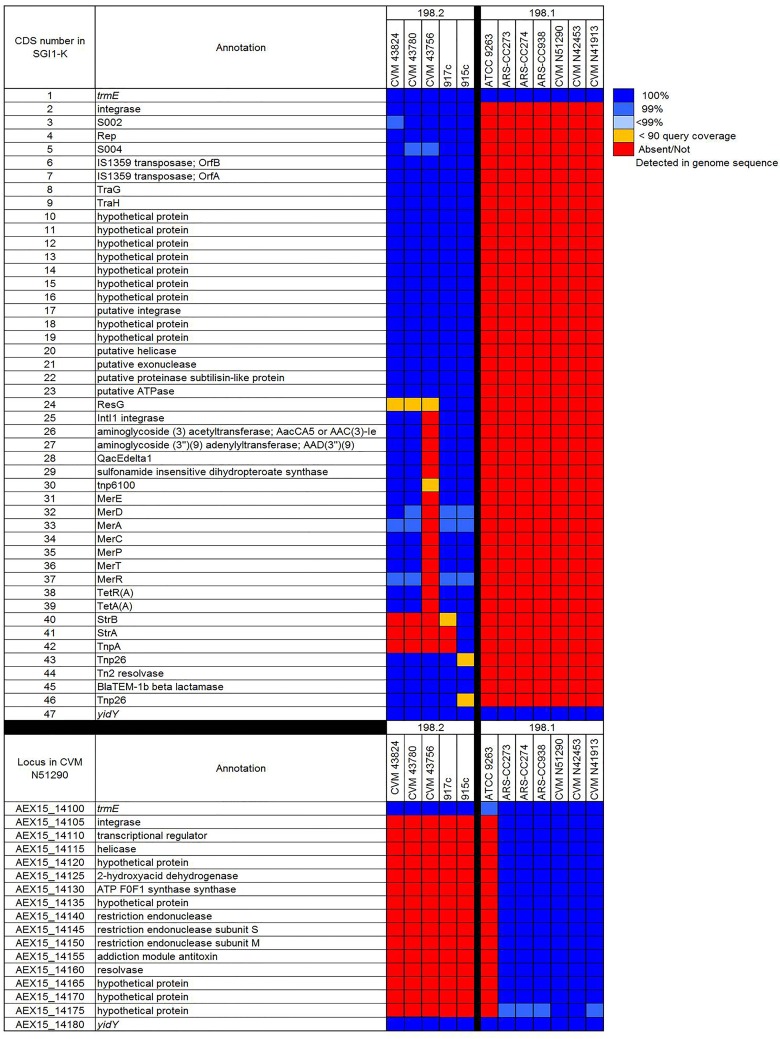
BLASTN comparison of SGI1-K against the ST198 genomes (Top). Blue squares indicate level of similarity, red squares indicate that homologs were not detected in the query genome sequence, orange squares = homolog was identified but at < 90% coverage. BLASTN comparison of a ca. 17 kb region inserted at the *trmE*-*yidY* locus in cluster 198.1 genomes (Bottom).

All cluster 198.2 genomes were identified as encoding ORFs homologous to the complete sequence of *Salmonella* Genomic Island 1 inserted at the *trmE*-*yidY* locus (SGI-K used as a reference; NCBI accession AY463797) (Fig 11). However, the structure of this island was difficult to discern due to the presence of SGI1 ORFs on multiple contigs in the 198.2 cluster genomes. SGI1 is an integrative and mobilizable element found in several *S*. *enterica* serovars and other non-salmonellae and responsible for resistance to multiple antibiotics [53][54]. The structure of this island is known to be highly variable [44][55]. Four of five SGI1 encoding genomes encoded regions known for resistance to mercury. However, this regions is absent from in CVM 43756 (Fig 11). For all cluster 198.2 genomes excluding 915c, *strB* (streptomycin phosphotransferase) and *strA* were not detected.

At the *trmE*-*yidY* insertion locus (AEX15_14100, AEX15_14180) all 198.1 genomes, excluding ATCC 9263, encoded a ca. 17 kb region with ORFs annotated as an integrase, transcriptional regulator, helicase, five ORFs annotated as hypothetical proteins, a single ORF annotated as 2-hydroxyacid dehydrogenase, ATP F0F1 synthase synthase, addiction module antitoxin, resolvase, and three restriction endonuclease ORFs. To-date, this region has only been identified in these six *S*. Kentucky ST198 genomes, *S*. Seftenberg ATCC 43845 and *S*. Agona strains 18.H.07, 557928, and 620239.

Based on the PlasmidFinder analysis, plasmids were not detected in any of the ST198 strains except for *S*. Kentucky CVM 51290 (Table 3). However, strains harboring plasmids carrying antibiotic resistance genes have been collected from human clinical cases [9] suggesting that, like ST152 and other *S*. *enterica* serovars, plasmid presence is variable in ST198. The IncI1 plasmid sequence identified in *S*. Kentucky CVM 51290 is similar to others currently in a NCBI plasmid database and shows 99% sequence similarity across 90% of the putative plasmid region with plasmid pSL476_91 from S. Heidelberg str. SL476, and 99% similarity across 88% of the plasmid region with *S*. Kentucky plasmid pCS0010A_95.

Few conclusions, based on genomic data, can be made about the ST198 group at this time due to the limited number of available *S*. Kentucky ST198 genome sequences deposited in public databases. However, it is clear that there is considerable genomic diversity among the genomes that have been sequenced to date. Sequencing of more ST198 genomes will allow for a more comprehensive understanding of the geophylogeny, global diversity, and presence of variable regions, such as SGI1, in these isolates.

It is important to note that ST198 has been isolated from avian and bovine sources in the United States [6] indicating that it may have the potential to become established in poultry flocks, dairy herds, other livestock, and/or wildlife in the United States. However, more work needs to be conducted to understand its apparent limited distribution in these animals as well as the presence of any potential wildlife reservoirs. Further, more work needs to be conducted to estimate the virulence potential of the ST198 that is endemic in the United States and abroad.

### Differences between *S*. Kentucky ST152 and ST198 Isolates

**Fig 12:**
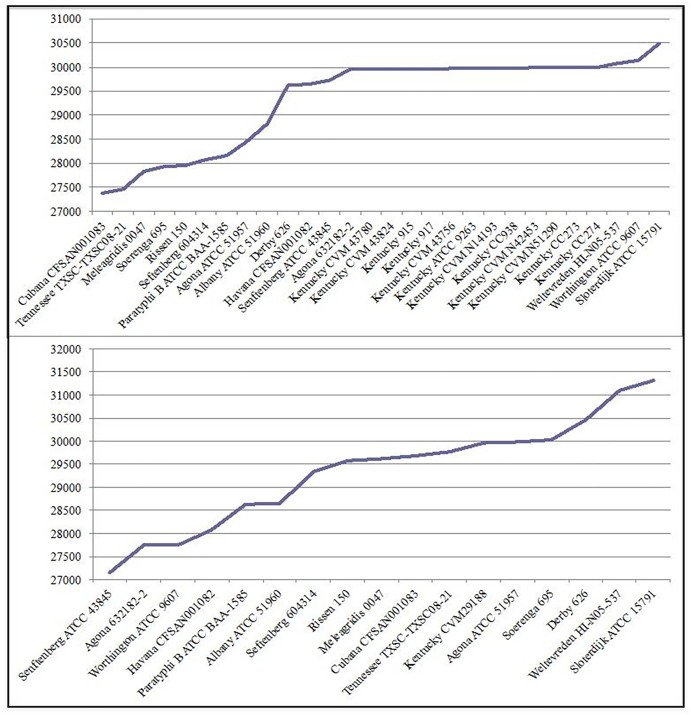
Number of SNP differences between *S*. Kentucky CVM29188 (ST152) and other *S*. *enterica* subclade A1 genomes (Top). Number of SNP differences between *S*. Kentucky ATCC 9263 (ST198) and other *S*. *enterica* subclade A1 genomes (bottom). X-axes show genomes used in this analysis and Y-axes show number of SNP differences.

Although frequently considered to be similar groups of strains due to their serological-based nomenclature, *S*. Kentucky ST198 and *S*. Kentucky ST152 isolates demonstrate considerable differences in their genetic backgrounds that have not been adequately described. Using assembled genomes, with the chromosome of *S*. Kentucky CVM29188 as a reference, there were between 27,369 and 30,485 SNP differences between *S*. Kentucky CVM29188 and representatives of other subclade A1 serovars in a core-genome alignment of length 3,465,932 bp (ca. 72% coverage of the *S*. Kentucky CVM29188 chromosome) (Table 6, Fig 12). In this analysis there were between 29,952 and 29,990 SNPs between CVM29188 and ST198 genomes. Thirteen serovars had fewer SNP differences with *S*. Kentucky CVM29188 than did *S*. Kentucky ST198 with CVM29188 (Table 6, Fig 12), while, 11 serovars of subclade A1 demonstrated fewer SNP differences with ST198 *S*. Kentucky ATCC 9263 than did *S*. Kentucky CVM29188 with *S*. Kentucky ATCC 9263. These data indicate a significant difference in the nucleotide sequence content between ST152 and ST198 genomes and coupled with the core-genome phylogeny (Fig 1) indicate that *S*. Kentucky ST152 and ST198 are more similar to other serovars than they are to each other.

**Table 6:** Number of SNP differences between ST198/ST152 and subclade A1 serovars. For this analysis representatives of subclade A1 were selected. Parsnp was used to identify core-genome SNPs among the sequenced isolates. The chromosome of *S*. Kentucky CVM29188 was used as a reference genome for the SNP analysis.

Serovar/Strain	No. SNP differences with *S*. Kentucky CVM29188		Serovar/Strain	No. SNP differences with *S*. Kentucky ATCC 9263
*S*. Cubana CFSAN001083	27369		*S*. Senftenberg ATCC 43845	27140
*S*. Tennessee TXSC-TXSC08-21	27457		*S*. Agona 632182–2	27743
*S*. Meleagridis 0047	27821		*S*. Worthington ATCC 9607	27746
*S*. Soerenga 695	27927		*S*. Havana CFSAN001082	28073
*S*. Rissen 150	27958		*S*. Paratyphi B ATCC BAA-1585	28638
*S*. Seftenberg 604314	28066		*S*. Albany ATCC 51960	28658
*S*. Paratyphi B ATCC BAA-1585	28156		*S*. Seftenberg 604314	29329
*S*. Agona ATCC 51957	28445		*S*. Rissen 150	29582
*S*. Albany ATCC 51960	28815		*S*. Meleagridis 0047	29614
*S*. Derby 626	29621		*S*. Cubana CFSAN001083	29685
*S*. Havana CFSAN001082	29635		*S*. Tennessee TXSC-TXSC08-21	29768
*S*. Senftenberg ATCC 43845	29718		*S*. Kentucky CVM29188 (ST152)	29971
*S*. Agona 632182–2	29940		*S*. Agona ATCC 51957	29974
*S*. Kentucky ATCC 9263 (ST198)	29971		*S*. Soerenga 695	30015
*S*. Weltevreden HI-N05-537	30078		*S*. Derby 626	30451
*S*. Worthington ATCC 9607	30140		*S*. Weltevreden HI-N05-537	31088
*S*. Sloterdijk ATCC 15791	30485		*S*. Sloterdijk ATCC 15791	31309

In an analysis comparing only ST152 genomes to ST198 genomes 37,769 SNP differences were detected (S3 Table). These SNPs were annotated and results demonstrated a significant number of polymorphisms in the protein-coding regions of the genome (S4 Table). Of a total of 4452 annotated protein-coding genes, at least one SNP was detected in 3713 (83.5%) genes while no SNPs were detected in 737 (16.5%) genes. Three hundred seventeen of these genes had ≥ 20 conserved SNP differences with ST152. Of particular interest are several protein coding genes that are involved with interactions with the animal host environment, specifically in the colonization and infection processes by *S*. *enterica*. For example, 239 conserved SNP differences were identified in SeKA_A3913, a 16,683 bp ORF annotated as a conserved hypothetical protein, located within SPI-4, and involved in colonization of the bovine gastrointestinal tract [56]. It should be noted that there was an appreciable level of sequence divergence in this gene within ST152 isolates, particularly in isolate ABB1087-1, and this is most likely due to the likelihood of synonymous substitutions to accumulate across a large protein coding gene. SeKA_A0255 of ST152, an ORF homologous to the secreted virulence protein SlrP (STM0800) of *S*. Typhimurium LT2, had 84 conserved SNP differences with ST198 genomes. A large repetitive protein (SeKA_A2250) homologous to BapA (STM2689) involved in host-colonization, organ invasion, and biofilm formation [57] encoded 68 conserved SNP differences between the two STs. An invasion-like protein (SeKA_A1129), homologous to STM1669 (ZirT), involved in virulence modulation [58] encoded 51 conserved SNP differences between the two STs. ShdA (SeKA_A1896), an outer-membrane protein that is expressed in the intestine and is involved in long-term fecal shedding [59][60], had 48 conserved SNP differences between the two STs. Multiple effector proteins also demonstrated appreciable divergence between ST152 and ST198 (SeKA_A1084 = *sseJ*; SeKA_A1053 = *sifB*; SeKA_A1621 = *sopA*; SeKA_A0837 = *sseC*, SeKA_A0835 = *sseB*; SeKA_A2465 = *sopD*; SeKA_A2283 = *pipB*; SeKA_A2398 = *sipD*; SeKA_A0839 = *sseE*; SeKA_A2393 = *sptP*; SeKA_A0841 = *sseF*; SeKA_A2380 = *avrA*). These proteins interact with host-cells and are intimately involved in the infection process [61][62][63] and their sequence divergence may result in different interactions in the human, cow, and poultry hosts, with the potential for different outcomes for each host. However, to fully assess the biological consequences of these differences *in vivo* analyses targeting these regions would need to be conducted.

Regions identified in one ST but absent in the other, as well as regions present in both but flanked by unique ORFs were identified by conducting a whole-genome reciprocal BLASTP analysis (Table 7). Sixteen regions of contiguous protein-coding genes present in ST198 genomes but absent in ST152 genomes, or highly diverged and flanked by unique regions were identified. Of these, six were completely or partially identified as genomic islands. Of particular interest in the ST198 genomes is an eight ORF region (region 3 in Table 7) responsible for sialic acid transport, a key feature of mammalian pathogens that allows them to scavenge sialic acid and utilize it as a carbon source in the host intestine (AEX15_07505, AEX15_07550) [64][65][66]. This region is flanked on one side by a tRNA-Ser locus suggesting it is a putative transmissible genomic island. A BLASTP analysis demonstrates there is a unidirectional match at low to moderate amino acid similarity (23 to 70% similarity) when these ORFs are compared to ST152 genomes indicating they are highly diverged from ORFs with a similar functional annotations encoded in the ST152 backbone. This sialic acid transport region was identified in other *S*. *enterica* serotypes, many of which are known pathogens of humans and other mammals, such as *S*. Typhimurium, *S*. Paratyphi A, *S*. Enteritidis, and *S*. Dublin. Another region of interest within the ST198 genomes consists of 15 ORFs involved inositol catabolism (region 4) (AEX15_17525, AEX15_17625). This region was also identified in several other serovars and has been identified as a myo-Inositol utilization island by Kröger and Fuchs [67]. Chaudhuri *et al*. [68] demonstrated this region as being associated with proliferation in the host gut. Further, *in vivo* studies have shown that salmonellae with an inactive *reiD* (the orphan regulator of the myo-inositol utilization island) demonstrated reduced fitness following oral infections or chickens, calf, and pigs [68]. *S*. Kentucky ST152 isolates, however, lack this island and, based on their prevalence in this environment, are suitably adapted for persistence in the bovine gut. A third region of interest is a nine ORF region identified within SPI-6 and encoding genes involved in alpha-fimbriae expression, a transcriptional regulator and a mobile genetic element (region 5) (AEX15_23130, AEX15_23170). This region is homologous to the Typhi-colonization factor (tcf) operon (STY0345-STY0348) and should be further investigated as a possible mechanism by which ST198 isolates colonize and infect human and animal hosts.

**Table 7:** Regions identified in one *S*. Kentucky ST (ST152 or ST198) and absent or partially present in the other.

ST	Region	Features	Size ^A^	Flanking Loci in ST198 (CVM N51290 or ATCC 9263) ^B^	Homologous Flanking Loci in ST152 (CVM29188)
198	1	histone acetyltransferase HPA2, hypothetical proteins	4405	AEX15_02835, AEX15_02865	SeKA_A3272, SeKA_A3273
	2	putative superfamily I DNA helicases, putative restriction endonuclease, putative type II restriction enzyme methylase subunit, putative cytoplasmic protein, putative inner membrane protein	24503	SEEK9263_16827, SEEK9263_t17079 (tRNA-Leu)	SeKA_A4142 (tRNA-Leu), SeKA_A4161
	3	sialic acid metabolism (transport) ^D^	9036	AEX15_07510, AEX15_07550 (tRNA-Ser)	SeKA_A0529, SeKA_A0537 (tRNA-Ser)
	4	inositol catabolism	23081	AEX15_17525, AEX15_17625	SeKA_A4089, SeKA_A4088
	5	SPI-6 [Typhi colonization factor (*tcf*) operon] ^D^	8399	AEX15_23130, AEX15_23170	SeKA_A4630, SeKA_A4631
	6	anaerobic dimethyl sulfoxide reductase chain A, B, C ^E^	13280	AEX15_10355, AEX15_10415 ^C^	SeKA_A1139, SeKA_A1140 ^C^
	7	DNA-binding protein, phosphorylase, nucleotidyltransferas, exonuclease ^D^	9323	AEX15_12395, AEX15_12455 (tRNA-Asn)	SeKA_A1557, SeKA_A1558 (tRNA-Asn)
	8	hypothetical protein (secretin domain/putative phage-related secreted protein)	2459	AEX15_09660, AEX15_09685 ^C^	SeKA_A0993, SeKA_A1001 ^C^
	9	carbon Starvation, D-gluconate and ketogluconates metabolism, Lactate utilization	5674	AEX15_10030, AEX15_10060	SeKA_A1070, SeKA_A1073
	10	SPI-13 with inserted iron reductase, FMN-dependent NADH-azoreductase, hypothetical proteins	3314	AEX15_04620 (tRNA-Phe), AEX15_04645	SeKA_A2676 (tRNA-Phe), SeKA_A2689
	11	hypothetical proteins, reverse transcriptase (adjacent to SPI-9) ^D^	4247	AEX15_21645/AEX15_21650, AEX15_21675 ^C^	SeKA_A2255 (tmRNA), SeKA_A2274 ^C^
	12	glycine biosynthesis, glycine and serine utilization, serine-glyoxylate cycle, serine biosynthesis, glutamine, glutamate, aspartate and asparagine biosynthesis, threonine and homoserine biosynthesis	3958	AEX15_19945, AEX15_19970	SeKA_A3752, SeKA_A3753
	13	probable integrase remnant	1050	AEX15_01875, AEX15_01895 (tRNA-Ser)	SeKA_A0372, SeKA_A0399 (tRNA-Ser)
	14	type I restriction-modification system, putative inner membrane protein, hypothetical protein	10325	AEX15_20690, AEX15_20730 ^C^	SeKA_A4188, SeKA_A4198 ^C^
	15	hypothetical protein	693	AEX15_14050, AEX15_14060	SeKA_A3461, SeKA_A3465
	16	hypothetical proteins, phage tail protein, oxidoreductase, transglycosylase ^E^	25556	AEX15_11375, AEX15_11530	SeKA_A1348, SeKA_A1377 ^C^
ST	Region	Features	Size ^A^	Flanking Loci in ST152 (CVM29188)	Homologous Flanking Loci in ST198 (CVM N51290 or ATCC 9263)
152	1	transcriptional regulator (LysR family) hypothetical protein, major facilitator superfamily MFS_1, 3-oxoacyl-[acyl-carrier-protein] reductase, transketolase, aldehyde-alcohol dehydrogenase 2	8040	SeKA_A0275, SeKA_A0283 ^C^	AEX15_01480, AEX15_01485
	2	Entner-Doudoroff pathway, glycolysis and gluconeogenesis, respiratory dehydrogenases 1, inositol catabolism, benzoate degradation ^D^	23239	SeKA_A0372, SeKA_A0399 (tRNA-Ser)	AEX15_01875, AEX15_01895 (tRNA-Ser)
	3	hypothetical proteins ^D^	3951	SeKA_A0529, SeKA_A0537 (tRNA-Ser)	AEX15_07510, AEX15_07550 (tRNA-Ser)
	4	hypothetical proteins, protein TolA	4683	SeKA_A0993, SeKA_A1001 ^C^	AEX15_09685, AEX15_09660 ^C^
	5	phage ORFs ^D^	33283	SeKA_A1348, SeKA_A1377 ^C^^,^ ^F^^,^ ^G^	AEX15_11530, AEX15_11375 ^C^
	6	hypothetical proteins, protein YibA, protein RhsD, IS1-family insertion element ^D^	4847	SeKA_A1701, SeKA_A1712	AEX15_13120, AEX15_13125
	7	hypothetical proteins, putative membrane protein, putative cytoplasmic proteins, transposases, HTH domain protein, integrase ^D^	11111	SeKA_A2096, SeKA_A2114 ^C^	AEX15_15380, AEX15_15440 ^C^
	8	prophage integrase, hypothetical proteins, nuclease-related domain family protein, protein YpjI, DNA repair protein, toxin-antitoxin system (adjacent to SPI-9) ^D^	12402	SeKA_A2255 (tmRNA), SeKA_A2274 ^C^	AEX15_21645/AEX15_21650, AEX15_21675 ^C^
	9	glycolysis and gluconeogenesis, mannitol Utilization	5553	SeKA_A2624, SeKA_A2631	AEX15_04405, AEX15_04410
	10	hypothetical protein, colicin-E7 immunity protein, bacteriophage integrase (fragments overlap with SPIs-8 and 13) ^D^	4943	SeKA_A2676 (tRNA-Phe), SeKA_A2689	AEX15_04620 (tRNA-Phe), AEX15_04645
	11	hypothetical proteins, putative autotransporter	5146	SeKA_A3310, SeKA_A3314	AEX15_02655, AEX15_02660
	12	hypothetical proteins	5073	SeKA_A3672, SeKA_A3678	AEX15_19610, AEX15_19615
	13	DNA phosphorothioation, hypothetical proteins, integrase ^D^	17034	SeKA_A4142 (tRNA-Leu), SeKA_A4161 ^C^	SEEK9263_t17079 (tRNA-Leu), SEEK9263_16827 ^C^
	14	type I restriction-modification system, putative inner membrane protein, hypothetical protein	10240	SeKA_A4188, SeKA_A4198	AEX15_20690, AEX15_20730
	15	oxidoreductase, inner membrane metabolite transport protein, sorbitol dehydrogenase, fructose-bisphosphate aldolase, kinase.	9227	SeKA_A0714, SeKA_A0725 ^C^	AEX15_08380, AEX15_08375 ^C^
	16	putative membrane transport protein, mandelate racemase/muconate lactonizing enzyme, putative transcriptional regulator	3481	SeKA_A3461, SeKA_A3465	AEX15_14060, AEX15_14050
	17	peroxisomal (S)-2-hydroxy-acid oxidase, putative glycolate oxidase	1202	SeKA_A1070, SeKA_A1073	AEX15_10030, AEX15_10060

^A^ = based on start/stop positions of proten encoding genes within region

^B^ = flanking loci

^C^ = encodes a string of homologous sequences but with unique flanking regions/may be unidirectional hits.

^D^ = putative genomic island by as determined by IslandViewer3 [41].

^E^ = part of this region predicted to be a genomic island by IslandViewer3 [41].

^F^ = not detected in ARS-CC7487

^G^ = truncated in CVM N45934

Seventeen contiguous regions of ST152 genomes were determined to be absent in ST198 genomes by a similar reciprocal BLASTP analysis. Eight of these regions were identified as putative genomic islands. Several of these regions were annotated as arrays of hypothetical proteins with no known function. Other islands encoded ORFs annotated as being involved in metabolic functions such as region 2 (ORFs involved in glycolysis and gluconeogenesis, inositol catabolism, benzoate degradation) (SeKA_A0372, SeKA_A0399), region 9 (ORFs involved in glycolysis and gluconeogenesis, mannitol utilization) (SeKA_A2624, SeKA_A2631), and region 15 (sorbitol dehydrogenase, fructose-bisphosphate aldolase) (SeKA_A0714, SeKA_A0725). The presence of these operons in strains frequently isolated from dairy cows and poultry suggests their roles in these environments should be further investigated.

In both STs ten homologous insertion loci flanking different protein encoding regions were identified, indicating that they may be hotspots for integration of laterally transferred DNA in *S*. *enterica*. Five insertion loci encoded tRNA sequences and one encoded a tmRNA sequence, which are known to be regions of genomic island insertion [69]. For the most part the ST-specific islands were conserved among all members of each ST indicating that they may play a significant role in the ecology of these strains and/or they are anchored in the genomes of either host ST.

## Conclusions

Based on the analysis conducted herein, there is a phylogenetic difference between poultry-associated and bovine-associated North American *S*. Kentucky ST152 isolates which is discernible due to four core-genome SNPs. However, several clusters of bovine-associated *S*. Kentucky ST152 isolates are more closely related to some poultry-associated isolates than they are to other bovine-associated isolates. This divergence is also associated, *in silico*, with the presence of several plasmids, one of which (ColV) has been demonstrated to enhance the colonization capacity of these strains in the chicken gastrointestinal tract, the other being a high frequency IncX1 plasmid with no known biological role. The influence of the core-genome SNPs and this IncX1 plasmid on the ecology of *S*. Kentucky ST152 in the bovine and poultry hosts, or survival in food sources or the environment should be further evaluated *in vivo*.

A significant difference in gene content and core-genome nucleotide sequence divergence between *S*. Kentucky ST152 and ST198 isolates was also observed. Based on the methods used in this study, both sequence types are phylogenetically more closely related to other serovars than they are to each other and their shared nomenclature stems from the high level of similarity between their antigenic coding regions which may have transferred laterally between sequence types. Several genomic elements in ST198, such as a sialic acid transport region, inositol catabolism and a homolog of the Typhi colonization factor (tcf), and differences in amino acid sequence of virulence-associated proteins may result in different interactions in the human, bovine, and poultry gastrointestinal tracts, an area that requires further research.

Although the predominant *S*. Kentucky strains recovered from both dairy cows and poultry in the United States do not appear to cause considerable disease in the animal hosts, the apparent high prevalence in these food animals represents a food safety risk to consumers of beef, dairy, and poultry products. Although infrequent, human clinical salmonellosis caused by *S*. Kentucky has been reported in the United States, but there is not much available data on the sequence types of human-clinical *S*. Kentucky in this country. The recent establishment of CIP^R^ *S*. Kentucky ST198 in poultry in France and Poland and other regions along with the detection of this ST in dairy cows, raw milk, ground beef, and ground turkey suggests the presence of this potential emerging pathogen should be monitored in food-producing animals.

## Supporting Information

S1 Table Genbank/SRA accession numbers for genomes sequenced for this study. (XLSX)

S2 Table Plasmids identified in each genome analyzed in this study. (XLSX)

S3 Table SNP differences between all *S*. Kentucky ST152 genomes and all *S*. Kentucky ST198 genomes. (XLSX)

S4 Table Annotation of SNPs in protein coding genes identified in an analysis of all *S*. Kentucky ST152 genomes versus all *S*. Kentucky ST198 genomes. (XLSX)
